# Deploying dengue-suppressing *Wolbachia*: robust models predict slow but effective spatial spread in *Aedes aegypti*

**DOI:** 10.1016/j.tpb.2017.03.003

**Published:** 2017-04-12

**Authors:** Michael Turelli, Nicholas H. Barton

**Affiliations:** a Department of Evolution and Ecology, University of California, Davis, California, United States of America; b Institute of Science and Technology, Am Campus 1, A-3400 Klosterneuburg, Austria

**Keywords:** population transformation, population replacement, bistable wave dynamics, disease suppression, Zika, biocontrol

## Abstract

A novel strategy for controlling the spread of arboviral diseases such as dengue, Zika and chikungunya is to transform mosquito populations with virus-suppressing *Wolbachia*. In general, *Wolbachia* transinfected into mosquitoes induce fitness costs through lower viability or fecundity. These maternally inherited bacteria also produce a frequency-dependent advantage for infected females by inducing cytoplasmic incompatibility (CI), which kills the embryos produced by uninfected females mated to infected males. These competing effects, a frequency-dependent advantage and frequency-independent costs, produce bistable *Wolbachia* frequency dynamics. Above a threshold frequency, denoted *p̂*, CI drives fitness-decreasing *Wolbachia* transinfections through local populations; but below *p̂*, infection frequencies tend to decline to zero. If *p̂* is not too high, CI also drives spatial spread once infections become established over sufficiently large areas. We illustrate how simple models provide testable predictions concerning the spatial and temporal dynamics of *Wolbachia* introductions, focusing on rate of spatial spread, the shape of spreading waves, and the conditions for initiating spread from local introductions. First, we consider the robustness of diffusion-based predictions to incorporating two important features of *w*Mel-*Aedes aegypti* biology that may be inconsistent with the diffusion approximations, namely fast local dynamics induced by complete CI (*i.e*., all embryos produced from incompatible crosses die) and long-tailed, non-Gaussian dispersal. With complete CI, our numerical analyses show that long-tailed dispersal changes wave-width predictions only slightly; but it can significantly reduce wave speed relative to the diffusion prediction; it also allows smaller local introductions to initiate spatial spread. Second, we use approximations for *p̂* and dispersal distances to predict the outcome of 2013 releases of *w*Mel-infected *Aedes aegypti* in Cairns, Australia, Third, we describe new data from *Aedes aegypti* populations near Cairns, Australia that demonstrate long-distance dispersal and provide an approximate lower bound on *p̂* for *w*Mel in northeastern Australia. Finally, we apply our analyses to produce operational guidelines for efficient transformation of vector populations over large areas. We demonstrate that even very slow spatial spread, on the order of 10-20 m/month (as predicted), can produce area-wide population transformation within a few years following initial releases covering about 20-30% of the target area.

## 1. Introduction

*Wolbachia* are maternally inherited endosymbionts, pervasive among arthropods (Weinert et al. 2015) and best known for reproductive manipulation (Werren et al. 2008). Their most widely documented reproductive manipulation is cytoplasmic incompatibility (CI) (Hoffmann and Turelli 1997; Hamm et al. 2014), which kills embryos produced by *Wolbachia*-uninfected females mated to infected males. *Wolbachia*-infected females are compatible with both infected and uninfected males and generally produce only infected progeny. CI gives infected females a reproductive advantage that increases with the infection frequency. Consequently, CI-inducing *Wolbachia* can spread within and among populations, at least once they become sufficiently common that the CI-induced advantage overcomes any frequency-independent disadvantages (Caspari and Watson 1959; Turelli and Hoffmann 1991; Turelli 2010; Barton and Turelli 2011). Because *Wolbachia* are maternally transmitted, selection favors variants that increase the fitness of infected females (Turelli 1994; Haywood and Turelli 2009). Teixeira et al. (2008) and Hedges et al. (2008) discovered that *Wolbachia*-infected individuals are protected from some pathogens, including viruses. Pathogen protection is not universal (Osborne et al. 2009), and studies of both transient somatic *Wolbachia* transinfections (Dodson et al. 2014) and stable transinfections (Martinez et al. 2014) suggest that *Wolbachia* can occasionally enhance susceptibility to pathogens. However, virus protection seems to be a common property of both natural and introduced *Wolbachia* infections (Martinez et al. 2014).

This anti-pathogen effect has revitalized efforts to use *Wolbachia* for disease control, an idea first proposed in the 1960s (Laven 1967; McGraw & O'Neill 2013). The disease-vector mosquito *Aedes aegypti* has been transinfected with two *Wolbachia* strains from *Drosophila melanogaster* (*w*MelPop, McMeniman et al. 2009; and *w*Mel, Walker *et al*. 2011). Two isolated natural Australian *Ae. aegypti* populations have been transformed with *w*Mel to suppress dengue virus transmission (Hoffmann et al. 2011), and these populations have remained stably transformed for more than four years (Hoffmann et al. 2014; S. L. O'Neill, pers. comm.). The dengue-suppressing phenotype of *w*Mel-transinfected *Ae. aegypti*, first demonstrated in laboratory colonies (Walker et al. 2011), has been maintained, and possibly enhanced, after two years in nature (Frentiu et al. 2014). Recently, *w*Mel has also been shown to block the spread of the Zika virus by *Ae. aegypti* (Dutra et al. 2016). *Anopheles stephensi* was also transinfected with *Wolbachia*, making them less able to transmit the malaria-causing parasite (Bian et al. 2013). *Wolbachia* transinfections are now being deployed for disease control in at least five countries (Australia, Vietnam, Indonesia, Brazil and Colombia, see the “Eliminate Dengue” website: http://www.eliminatedengue.com/program), with many more releases planned. We present simple approximation-based predictions to understand and aid the deployment of these transinfections.

Our mathematical analyses rest on bistable frequency dynamics for *Wolbachia* transinfections. Namely, the frequency-independent costs associated with introduced infections cause frequencies to decline when the infections are rare; but the frequency-dependent advantage associated with CI overcomes these costs when the infections become sufficiently common. As explained in the Discussion, bistability now seems implausible for naturally occurring *Wolbachia* infections (cf. Fenton et al. 2011; Kriesner et al. 2013; Hamm et al. 2014). However, we present several lines of evidence, including new field data, indicating that *w*Mel transinfections in *Ae. aegypti* experience bistable dynamics in nature.

Why does bistability matter? As reviewed in Barton and Turelli (2011), bistability constrains which variants can spread spatially, how fast they spread, how difficult it is to initiate spread, and how easily spread is stopped. Roughly speaking, spatial spread can occur only if the critical frequency, denoted *p̂*, above which local dynamics predict deterministic increase rather than decrease, is less than a threshold value near ½. As discussed in Turelli (2010), *p̂* is determined by a balance between the frequency-dependent advantage provided by cytoplasmic incompatibility and frequency-dependent disadvantages associated with possible deleterious *Wolbachia* effects on fecundity, viability and development time. As *p̂* increases, the rate of predicted spatial spread slows to zero (then reverses direction), the area in which the variant must be introduced to initiate spread approaches infinity, and smaller spatial heterogeneities suffice to halt spread. Spatial dynamics depend on details of local frequency dynamics and dispersal that are not well understood empirically. This motivates our exploration of quantitative predictions using relatively simple but robust models that focus on three key biological phenomena, dispersal, deleterious fitness effects and cytoplasmic incompatibility.

We seek conditions under which minimal releases of dengue-suppressing *Wolbachia* transinfections achieve area-wide disease control by transforming a significant fraction of the vector population in a relatively short period. We focus on simple models to provide quantitative predictions and guidelines, and test the robustness of the predictions to long-distance dispersal. Our simple approximations make testable predictions that may be improved as additional data become available. Many parameters in detailed models will be difficult to estimate and are likely to vary in time and space. Our idealization is motivated by the scarce information concerning the ecology of disease vectors such as *Ae. aegypti*. For instance, the dynamics of introductions must depend on ecological factors such as density regulation (Hancock et al. 2011a,b). However, the ecology *of Ae. aegypti* is so poorly understood that increases in embryo lethality associated with CI might lead to either decreasing or increasing adult numbers (cf Prout 1980; Walsh et al. 2013; but see Hancock et al. 2016). As in Barton and Turelli (2011), we ignore these ecological complications and emphasize quantitative conclusions that depend on only two key parameters: *σ*, a measure of average dispersal distance, and *p̂*, the unstable equilibrium frequency. We illustrate how these two parameters can be estimated from in*troduced-Wolbachia* frequency data (producing predictions that can be cross-validated) and explore the robustness of the resulting predictions.

Our new analyses build on Barton and Turelli (2011), which used diffusion approximations to understand spatial and temporal dynamics. To determine the robustness of those diffusion-based predictions, which make mathematical assumptions that may not be consistent with the biology of *Wolbachia*-transinfected mosquitoes, we examine dispersal patterns that assign higher probabilities to long-distance (and very short-distance) dispersal. We ask how dispersal patterns affect wave speed, wave shape, and the conditions for initiating an expanding wave (Section 4). We use these new, more robust predictions to propose guidelines for field deployment of dengue-suppressing *Wolbachia*. This involves addressing new questions. For instance, Barton and Turelli (2011) determined the minimum area over which *Wolbachia* must be introduced to initiate spatial spread, but ignored the fact that near this critical size threshold, dynamics would be extremely slow. Effective field deployment requires initiating multiple waves to cover a broad area relatively quickly, given constraints on how many mosquitoes can be released. This requires understanding how transient dynamics depend on initial conditions. We synthesize data-based and model-based analyses of spatial spread to outline efficient strategies for area-wide transformation of vector populations (Section 7).

In addition to our theoretical results concerning predicted properties of spatial spread and near-optimal release strategies, we illustrate the theory with predictions concerning the outcome of wMel releases in Cairns, Australia in 2013 (Section 5). We also analyze some previously unpublished data from the 2011 releases reported in Hoffmann et al. (2011) to approximate a lower bound for *p̂* relevant to the Cairns releases (Section 6).

## 2. Mathematical background, models and methods

Our initial numerical analyses focus on testing the robustness of predictions presented in Barton and Turelli (2011). We first describe the diffusion approximations and results from Barton and Turelli (2011) before describing the alternative approximations and analyses. Next we describe the model used to analyze the new data we present. Finally we describe our approaches to approximating optimal release strategies.

### 2.1. Diffusion approximations, alternative dynamics and predictions

The simplest spatial model relevant to understanding *Wolbachia* frequency dynamics in space and time is a one-dimensional diffusion approximation:

(1) $$\frac{\partial p}{\partial t}=\frac{\sigma^{2}}{2}\frac{\partial^{2}p}{\partial x^{2}}+f(p),$$

where *f*(*p*) describes local dynamics and *p*(*x*, *t*) denotes the infection frequency at point *x* and time *t*, and *σ* denotes the standard deviation of dispersal distances. If we approximate the local bistable dynamics by the cubic

(2) $$f(p)=s_{h}(1-p)(p-\hat{p}),$$

where *s*_h_ describes the intensity of CI, there is an explicit asymptotic traveling wave solution of (1), given as Eq. 13 of Barton and Turelli (2011). Eq. (1), extended to two dimensions as described by Eq. 22 of Barton and Turelli (2011), can be solved numerically to address transient dynamics associated with local releases. In two dimensions, we interpret *σ*^2^ as the variance in dispersal distance per generation along any axis. (This implies that the average Euclidean distance between the birthplaces of mothers and daughters is $\sigma \sqrt{\pi /2}$, assuming Gaussian dispersal.) The model defined by (1) and (2) provides analytical predictions for wave speed and wave shape and numerical conditions for establishing a spreading wave from a local introduction.

To more accurately approximate CI dynamics, Barton and Turelli (2011) replaced the cubic approximation (2) with the model of Schraiber et al. (2012)

(3a) $$\frac{dp}{dt}=f(p)=\frac{s_{h}p(1-p)(p-\hat{p})}{1-s_{f}p-s_{h}p(1-p)},\text{with}$$

(3b) $$\hat{p}=s_{r}/s_{h}=(s_{f}+s_{v}-s_{f}s_{v})/s_{h}.$$

(Eq. 3a assumes that the daily death rate for the infected individuals is *d*_I_ = 1, so that time is measured in terms of the average lifetime of an infected individual.) As in Eq. (2), *s*_h_ measures the intensity of CI; whereas *s_r_* measures the net reduction in fitness caused by the *Wolbachia* infection. As in the discrete-time model of Turelli (2010), fitness costs may involve reductions of both fecundity and mean life length (viability), as measured by *s*_f_ and *s*_v_, respectively; however, *s*_v_ enters the dynamics only through *p̂*. Numerical integration can be used to compare the cubic-based analytical predictions with those produced by this more biologically explicit approximation. For fixed *p̂*, the dynamics described by Eq. (3a) depend on whether fitness costs primarily involve viability or fecundity effects (because only *s_f_* appears in the denominator). The data of Hoffmann et al. (2014) suggest that fecundity effects may dominate.

In our discrete-time, discrete-space analyses, we approximate local dynamics with the Caspari-Watson model (1959) which incorporates CI and fecundity effects (cf Hoffmann and Turelli 1988; Weeks et al. 2007). Let *H* denote the relative hatch rate of embryos produced from an incompatible cross. Setting *H* = 1 – *s*_h_ and *F* = 1 – *s*_f_, and letting *p* denote the frequency of infected adults, the local dynamics are described by

(4a) $$\Delta p=p^{\prime }-p=\frac{s_{h}p(1-p)(p-\hat{p})}{1-s_{f}p-s_{h}p(1-p)},\text{with}$$

(4b) $$\hat{p}=s_{f}/s_{h}.$$

In this model, the condition for bistability (i.e., simultaneous local stability of *p* = 0 and *p* = 1) is *s*_h_ > *s*_f_ > 0, i.e., the (frequency dependent) benefit to the infection from CI must exceed its (frequency independent) cost, modeled as decreased fecundity. Both lab and field experiments indicate that *w*Mel causes complete CI, i.e., *s*_h_ ≈ 1 in *Ae. aegypti* (Hoffmann et al. 2014).

#### 2.1.1. Wave speed

Measuring time in generations, the predicted wave speed from (1) with cubic dynamics (2) is

(5) c=σsh(12−p^),

provided that *p̂* > 0. This one-dimensional result also describes the asymptotic speed of a radially expanding wave in two dimensions (see Eqs. 23-25 of Barton and Turelli 2011). Barton and Turelli (2011) used numerical solutions of (1) to compare this speed prediction to the wave speed produced by (3), which explicitly models the fast local dynamics associated with strong CI. The more realistic dynamics (3) led to slightly faster wave propagation (as expected because the denominator of *f*(*p*) is less than one).

#### 2.1.2. Wave width

The explicit traveling-wave solution of (1) for cubic *f*(*p*) provides a simple description for the asymptotic wave width, the spatial scale over which infection frequencies change. Defining wave width as the inverse of the maximum slope of infection frequencies (Endler 1977), the diffusion approximation with cubic dynamics implies that the traveling wave has width

(6) $$w=1/\mathrm{Max}(|\partial p/\partial x|)=4\sigma /\sqrt{s_{h}},$$

which becomes 4*σ* with complete CI, as in *Ae. aegypti*. The explicit solution that produces (6) implies that with *s*_h_ = 1, the scaled wave (with space measured in units of *σ*) has shape 1/[1 + *Exp*(−*x*)]. Thus, infection frequencies increase from about 0.18 to 0.82 over 3*σ*. If steady spread is observed in the field, we can use this wave-shape approximation to estimate *σ* from spatial infection-frequency data. These estimates can be compared to independent estimates from release-recapture experiments or genetic data. We show below that relation (6) is relatively robust to more realistic descriptions of local frequency dynamics and long-tailed dispersal.

#### 2.1.3. Wave initiation

Finally, the diffusion approximation predicts the minimum area that must be actively transformed to initiate deterministic spatial spread. Barton and Turelli (2011) consider introductions over a circular area with initial infection frequency *p*_0_ in the circle. This initial state corresponds to rapid local establishment of a transinfection from intensive releases. Hoffmann et al. (2011) showed that releases in isolated suburbs near Cairns produced *w*Mel frequencies over 80% within 12 weeks, about three *Ae. aegypti* generations. Fig. 3 of Barton and Turelli (2011) summarizes the diffusion predictions concerning the minimum radius of release areas, measured in units of dispersal distance *σ*, needed to initiate a spreading wave. In their analysis, the scaled critical radius, denoted *R*_crit_, depends only on *p̂*). As *p̂* increases from 0 to 0.3, *R*_crit_ increases from 0 to about 3.5σ, then rapidly increases towards infinity as *p̂* approaches 0.5, the approximate upper bound on *p̂* consistent with spatial spread. Releases over areas smaller than *R*_crit_ are predicted to fail, with the infection locally eliminated by immigration from surrounding uninfected populations. Barton and Turelli (2011) used the Schraiber et al. (2012) model (3) to assess the robustness of these cubic-based predictions to more realistic local CI dynamics. Model (3) produced *R*_crit_ predictions within a few percent of those derived from the cubic (see Fig. 3 of Barton and Turelli 2011), assuming that *Wolbachia* reduce fitness primarily through viability effects. Below, we contrast the diffusion predictions of Barton and Turelli (2011) with numerical results that account for fecundity effects, faster local dynamics and alternative forms of dispersal.

#### 2.1.4. Time scale for asymptotic wave speed and width

**Fig. 1:**
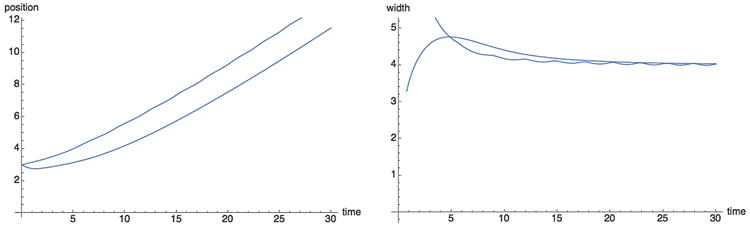
Transient dynamics of wave position and wave width from numerical solutions of the two-dimension version of diffusion model (1) with cubic dynamics (2), *s*_h_ = 1 (i.e., complete CI) and *p̂* = 0.25. The calculations assume circular introductions with radial symmetry and initial frequency *p*(*R*) = 0.8/{1 + exp[4(*R* – 3)/*v*]} for *v* = 0.8 and 8. (Note that *v* is a scale factor that determines the steepness with which infection frequency falls.) Setting *v* = 0.8 produces an abrupt drop in the initial frequency from 0.75 to 0.05 over roughly *R* = 2.5 to *R* = 3.5; with *v* = 8, the initial frequency drops from 0.65 at *R* = 0 to 0.25 at *R* = 4.6. The left panel shows wave position measured as the point of maximum slope, the right panel shows the width, measured as the inverse of the maximum slope (see Eq. (6)). The dotted curves correspond to *v* = 0.8, modeling a rapid introduction in a confined area. This produces a faster approach to the expected asymptotic speed of 0.25. Both initial conditions, one with narrower width than the asymptotic value of four, the other wider, approach the asymptotic width of four within 7-10 generations.

Predictions (5) and (6) for wave speed and wave width are based on the asymptotic behavior of the traveling wave solutions to the diffusion model (1) assuming cubic dynamics. (As discussed in Barton and Turelli (2011), the asymptotic wave speed and width are the same in one dimension and two.) To apply these predictions to frequency data generated from field releases, it is important to know how quickly these asymptotic values are approached. Fig. 1 illustrates numerical solutions for the transient dynamics of the cubic-diffusion model in two dimensions with plausible parameter values for *wM*el in *Aedes aegypti*, *s*_h_ = 1 and *p̂* = 0.25 (discussed below). The calculations use two initial conditions. In the first, the infection is introduced with *p*_0_ near 0.8 over a circular region with diameter 3 (see Fig. 1 legend for details), which is about 14% larger than *R*_crit_ = 2.64, the critical radius needed to initiate spatial spread. In the second, the infection frequency drops smoothly from 0.65 at the center of the introduction to 0.25 (the unstable point, *p̂*) at about *R* = 4.6. As shown in Fig. 1, for these parameters and initial conditions, the approach to the asymptotic wave speed and width is rapid, on the order of five-to-ten generations. Similar results hold for our discrete-time models and data from field releases of *w*Mel in *Aedes aegypti* (data not shown).

### 2.2. Numerical analyses of discrete-time, discrete-state (DTDS) models

To explore the robustness of the diffusion predictions, we consider the simultaneous effects of fast local dynamics, associated with complete CI, and long-tailed dispersal. To do this, we replace the PDE approximation (1) with discrete-time, discrete-space (DTDS) models that assume discrete generations and discrete patches in which the consequences of mating, fecundity effects and CI occur and between which adult migration occurs.

#### 2.2.1. Model structure and dynamics

Let *i* denote a patch in one or two dimensions, let *g*(*p*) = *p*′ denote a function that describes how mating, fecundity differences and CI transform local infection frequencies between generations, and let *m*(*i*, *j*) denote the probability that an individual at location *i* after migration originated in location *j*. Assuming discrete generations in which migration of newly eclosed individuals precedes local CI dynamics, the infection frequencies among adults in each patch follow

(7) $$p(i,t+1)=\sum_{j}m(i,j)p^{\prime }(j,t)\;\text{or}\;p(i,t+1)=g[\sum_{j}m(i,j)p(j,t)].$$

Our choice of patch spacing for these discretizations is discussed below. We approximate local dynamics with the Caspari-Watson model (4).

#### 2.2.2. Alternative dispersal kernels

Following Wang et al. (2002), we compare results obtained with three models of dispersal: a Gaussian, denoted *G*(*x*), versus two “long tailed” distributions, the Laplace (or reflected exponential), denoted *L*(*x*), and the exponential square root (ExpSqrt), denoted *S*(*x*). Letting *σ* denote the standard deviation of dispersal distances, our dispersal kernels in one dimension (proportional to the probability of moving distance *x* in one dimension) are

(8a) $$G(x)=\text{Exp}[-x^{2}/(2\sigma^{2})]/\sqrt{2\pi \sigma^{2}},$$

(8b) $$L(x)=\text{Exp}[-\sqrt{2x^{2}/\sigma^{2}}]/\sqrt{2\sigma^{2}},\text{and}$$

(8c) $$S(x)=\sqrt{15/(2\sigma^{2})}\text{Exp}[-\sqrt[{4}]{120x^{2}/\sigma^{2}]}.$$

**Fig. 2:**
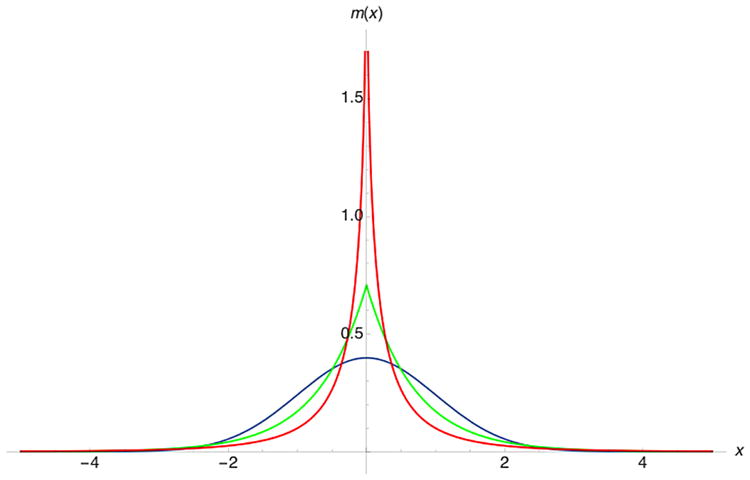
Alternative dispersal models with *σ* = 1. The three models are: Gaussian (blue), Laplace (green), and ExpSqrt (red) as described by (8). Each describes the probability, denoted *m*(*x*) in the figure, of moving distance *x* along any axis.

These alternative dispersal models are illustrated in Fig. 2. Our DTDS calculations used patch spacing of 0.5*σ* We truncated the dispersal functions at ±10*σ*. We adjusted the variance parameter in our discrete calculations so that the actual standard deviation of the discrete distribution was *σ*. In two dimensions, (*x*, *y*), the dispersal models, generally denoted *m*(*z*), were implemented as $m(\sqrt{x^{2}+y^{2}})$.

Taking logs of the densities, the tails of *G*(*x*) decline as −*x*^2^, whereas the tails of *L*(*x*) and *S*(*x*) decline as −|*x*| and $-\sqrt{\left|x\right|}$, respectively, corresponding to successively higher probabilities of long-distance dispersal. Denoting the random variables corresponding to these densities as *G*, *L* and *S*, we have *P*(|*G*| > 3*σ*) = 0.003, *P*(|*L*| > 3*σ*) = 0.014 and *P*(|*S*| > *3σ*) = 0.022. As Fig. 2 shows, higher probabilities of long-distance dispersal are accompanied by higher probabilities of short-distance dispersal (e.g., *P*(|*G*| < 0.5*σ*) = 0.383, *P*(|*L*| < 0.5*σ*) = 0.507 and *P*(|*S*| < 0.5*σ*) = 0.678), with corresponding medians for |*G*|, |*L*| and |*S*| of 0.67*σ*, 0.49*σ* and 0.28*σ*, respectively.

In two dimensions, we assume that dispersal is isotropic, with radial distribution given by the kernels defined by (8), and scaled such that the standard deviation along any one axis is *σ*. To approximate *σ* from experiments that estimate mean Euclidean dispersal distances, *D*, note that the Gaussian produces $E(D_{G})=\sigma \sqrt{\pi /2}\approx 1.25\sigma$, whereas for the Laplace and ExpSqrt, we have *E*(*D*_L_) ≈ 1.15*σ* and *E*(*D*_S_) ≈ 0.94*σ*, respectively. Thus, empirical estimates of average Euclidean dispersal distance can imply values of *σ* that differ by over 30% depending on the shape of the dispersal function, with longer tails implying higher values of *σ*. (Note that statistical estimation of dispersal requires some assumption about the distribution of dispersal distance.) We compare the predictions resulting from alternative dispersal models by holding fixed the variance parameter *σ*^2^, which is natural measure of dispersal distance for diffusion approximations (see, for instance, the derivations in Haldane (1948), Slatkin (1973) or Nagylaki (1975)).

### 2.3 Model used for data analysis: an isolated deme subject to immigration

Barton and Turelli (2011) adapted the “island model” of Haldane (1930) to approximate the rate of immigration of *Wolbachia*-infected individuals required to “flip” an isolated population from uninfected to infected. In addition to approximating the critical migration rate, *m*, the analysis produces an analytical approximation for the equilibrium infection frequency when the immigration rate is too low to flip the recipient population to *Wolbachia* fixation. Assuming complete CI, 100% frequency of *Wolbachia* in the donor population and one-way immigration into the recipient population, Eq. (31) of Barton and Turelli (2011) predicts that *Wolbachia* should take over the recipient population if *m*, the fraction of individuals who were new migrants each generation, exceeds m* = *p̂*^2^/4. For *m* < m*, the predicted *Wolbachia* equilibrium frequency in the recipient population, using a cubic approximation for local dynamics, is

(9) $$p*=(\hat{p}/2)-\sqrt{{\left(\hat{p}/2\right)}^{2}-m}<\hat{p}/2.$$

(this is a reparameterization of Eq. (31) of Barton and Turelli 2011). Hence, if we use the long-term average *Wolbachia* frequency, *p̄*, to approximate p*, the equilibrium described by (9), we can approximate a lower bound for the unstable equilibrium, *p̂*, as 2*p̄*.

### 2.4. Near-optimal release strategies

We analyze alternative release strategies using a combination of numerical solutions of diffusion models, DTDS models and even simpler models that assume constant rates of radial spread from release foci. Each analysis is described below along with the results it produces.

## 3. New data demonstrating bistability

We analyze a small subset of the *Wolbachia* infection frequency data collected subsequent to the first “Eliminate Dengue” field releases of *w*Mel-infected *Ae. aegypti*, described in Hoffmann et al. (2011). The releases occurred in early 2011 in two isolated towns, Gordonvale (668 houses) and Yorkeys Knob (614 houses), near Cairns in northeast Australia. As described in Hoffmann et al. (2011), Pyramid Estate (PE) is an area of Gordonvale separated from the town center by a major highway, with roughly 100 m separating the nearest houses on either side. Highways seem to inhibit *Ae. aegypti* migration (Hemme et al. 2010). The 2011 *w*Mel releases were restricted to the main part of Gordonvale; but as reported in Hoffmann et al. (2011), *w*Mel-infected mosquitoes were found in PE within months of the initial releases. The PE capture sites were scattered over an area of houses on the order of 1 km^2^ with traps roughly 100-500 m from the nearest residences in the Gordonvale release area. As described in Hoffmann et al. (2011, 2014), infection frequencies were estimated using PCR of DNA from adults reared from eggs collected in oviposition traps. Between late March 2011 and January 2015, 2689 adults were assayed in PE. The data are archived in Dryad (http://XXX).

## 4. Results: Robustness of diffusion results to long-tailed dispersal and rapid CI dynamics

### 4.1. Wave speed

**Fig. 3:**
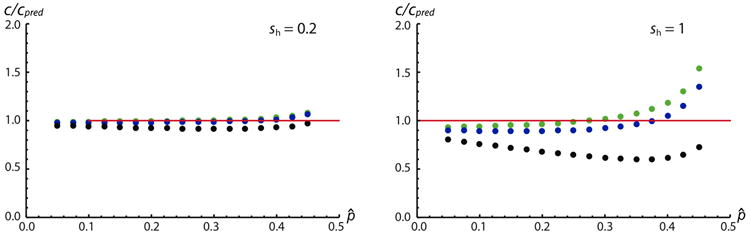
Wave speed. Speed calculated from DSDT analyses compared to the cubic-based diffusion prediction, c=σsh(12−p^), as a function of *p̂*, for *s*_h_ = 0.2 (left) and *s*_h_ = 1 (right). As in (2), *σ* denotes the standard deviation of dispersal distances; *s*_h_ denotes the intensity of CI. The green dots were produced with Gaussian dispersal, blue with Laplace and black with Exponential Square root (ExpSqrt).

We initially calculated wave speed in a one-dimensional spatial array, then as in Barton and Turelli (2011), we checked the results with two-dimensional calculations. To disentangle the effects of non-Gaussian dispersal from the effects of fast local dynamics, we contrast results obtained assuming complete CI (*s*_h_ = 1), as observed with *w*Mel-infected *Ae. aegypti*, with results assuming weak CI (*s*_h_ = 0.2). Fig. 3 compares the numerically approximated wave speeds to the analytical prediction, c=σsh(12−p^), from the diffusion approximation with cubic dynamics. The left panel shows that with relatively slow local dynamics (*s*_h_ = 0.2), the cubic diffusion approximation is accurate and robust to the shape of the dispersal function. This is expected from the derivation of approximation (1) as a limit of discrete-time, discrete-space dynamics (Haldane 1948; Nagylaki 1975). The derivation explicitly invokes slow local dynamics and limited dispersal, retaining only the variance of dispersal distances in the quadratic approximation. The *s*_h_ = 0.2 results have two other notable features. First, despite the overall accuracy of approximation (5), we see that ExpSqrt dispersal slightly slows propagation. This can be understood in terms of the lower median dispersal distance and the fact that with bistability, rare long-distance dispersal is not effective at pushing the wave forward, because long-distance migrants are swamped by the much more abundant natives. This distinguishes bistable spatial dynamics from those with zero as an unstable equilibrium. For such systems, long-tailed dispersal can produce accelerating waves (see Supporting Information Appendix A for references and comparison of bistable versus Fisherian wave speeds). Moreover, geographic spread associated with spatially non-contiguous, successful long-distance colonization events (cf Shigesada and Kawasaki 1997, Ch. 5), can greatly exceed predictions based on average dispersal distances. Second, note that as *p̂* approaches 0.5, the analytical approximation starts to underestimate wave speed. As described by Barton and Turelli (2011), this reflects the fact that the cubic model produces the threshold *p̂* ≤ 0.5 for spatial spread, whereas models more accurately describing CI and fitness costs, such as (3) and (4), predict spatial spread with *p̂* slightly above 0.5.

The right panel of Fig. 3 (*s*_h_ = 1) shows that faster local dynamics accentuate both phenomena seen with *s*_h_ = 0.2: slower speed with more long-tailed dispersal and underestimation of observed speed as *p̂* approaches 0.5. With complete CI and plausible *p̂* (*i.e.*, 0.2 ≤ *p̂* ≤ 0.35), observed speed closely follows the cubic-based diffusion prediction with Gaussian dispersal, but is reduced by about 10% for Laplace dispersal and by much more (25-40%) for ExpSqrt dispersal. A simple interpretation is that long-tailed dispersal is associated with smaller median dispersal distances. Long-distance migrants are effectively “wasted” in that they cannot initiate local spread.

Appendix A provides a more complete description of the consequences of alternative dispersal models on wave speed under bistable versus monostable local dynamics, including the consequences of finite population size at the front on wave propagation.

### 4.2. Wave width

**Fig. 4:**
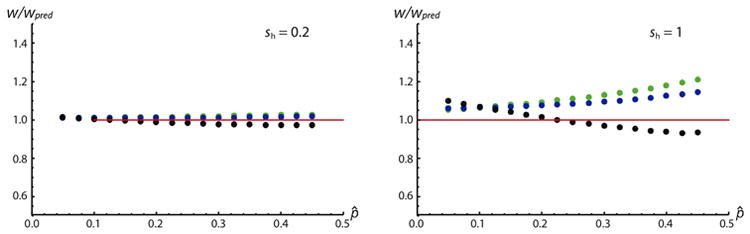
Wave width. Wave width as a function of *p̂* calculated using discrete-time, discrete-space (DTDS) analyses with alternative dispersal models. The dots from the DTDS analyses are compared to the diffusion prediction ( $w=1/\mathrm{Max}(|\partial p/\partial x|)=4\sigma /\sqrt{s_{h}}$, red line) for *s*_h_ = 0.2 (left) and *s*_h_ = 1 (right). As in Fig. 3, *σ* denotes the standard deviation of dispersal distances and *s*_h_ denotes the intensity of CI. The green dots were produced with Gaussian dispersal, blue with Laplace and black with Exponential Square root (ExpSqrt).

Under the diffusion model with cubic dynamics, the predicted wave width is $w\approx 4\sigma \sqrt{s_{h}}$(6). Fig. 4 compares this prediction with the results obtained from DTDS with Caspari-Watson dynamics and alternative dispersal models. With relatively slow local dynamics (*s*_h_ = 0.2), Panel A shows that the cubic-diffusion prediction remains accurate for all three dispersal models, analogous to the results for wave speed illustrated in Fig. 3A. ExpSqrt dispersal slightly reduces wave width, presumably reflecting the lower median dispersal. As with wave speed, *s*_h_ = 1 produces larger departures from the cubic-diffusion prediction and much greater effects of dispersal shape. However, for plausible values of *p̂* (*i.e*., 0.2 ≤ *p̂* < 0.35), the observed width remains within about 15% of prediction (6) for Gaussian and Laplace and very close to the prediction for ExpSqrt.

### 4.3. Wave initiation: critical radius R_crit_

**Fig. 5:**
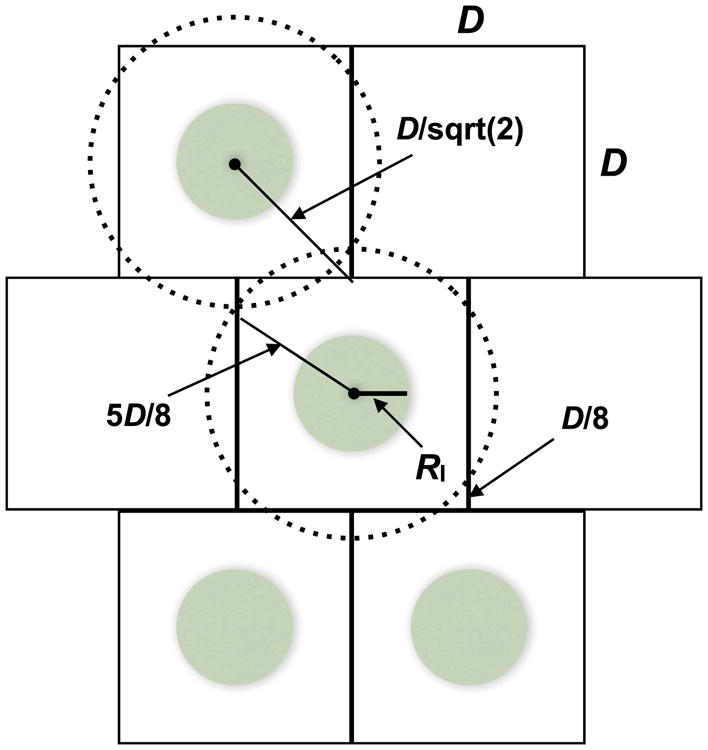
Critical radius, *R*_crit_, assuming complete CI and alternative dynamics. The upper points reproduce the diffusion results from Barton and Turelli (2011) with cubic (red curve) and Schraiber et al. (2012) CI dynamics (assuming only viability fitness costs). The red curve shows the cubic-diffusion predictions with initial infection frequency *p*_0_ = 0.8, the large blue dots are the cubic with *p*_0_ = 0.6. The small red (blue) dots are produced by the diffusion analysis of Schraiber et al. (2012) CI dynamics with *p*_0_ = 0.8 (*p*_0_ = 0.6). The lower points and curves show our DTDS predictions as a function of *p̂* and *p*_0_ with alternative dispersal models. The lower lines correspond to *p*_0_ = 0.8 with Gaussian (green), Laplace (blue) and ExpSqrt (black) dispersal. The points above and below these lines correspond to *p*_0_ = 0.6 and *p*_0_ = 1, respectively.

Fig. 3 of Barton and Turelli (2011) showed how *R*_crit_, the minimal radius of an introduction needed to initiate spread (measured in units of the dispersal parameter *σ*), depends on *p̂* and *p*_0_ under the diffusion approximation. It contrasts the predictions for cubic dynamics versus Schraiber et al. (2012) *Wolbachia* dynamics (Eq. 3). Fig. 5 compares those predictions to DTDS results under Caspari-Watson *Wolbachia* dynamics (Eq. 4). The key result is that long-tailed dispersal produces smaller critical radii, and the effect of long-tailed dispersal increases as *p̂* increases. This result is complementary to the wave-speed results. With longer-tailed dispersal, more individuals move very little so that the median dispersal falls, making it easier to establish a wave (but the resulting wave moves more slowly). The discrepancies between the diffusion results with Schraiber et al. (2012) dynamics and the DTDS results for Gaussian dispersal are mainly attributable to the fact that the Schraiber et al. (2012) results illustrated in Fig. 5 assume only viability costs, which produces slower dynamics (see Eq. 4a) and requires larger introductions, than if one assumes fecundity costs, as done in the DTDS Caspari-Watson model. The effect of fecundity vs. viability costs is illustrated in Table 1 in section 5.1. For *p̂* = 0.35 and *p*_0_ = 0.8, numerical solution of the diffusion equation with Schraiber et al. (2012) dynamics produces *R*_crit_ = 3.36 if *s*_f_ = 0 (so that *p̂* = *s*_v_), but this drops to *R*_crit_ = 2.76 if *s*_v_ = 0 (so that *p̂* = *s*_f_). The corresponding values under the DTDS model with Caspari-Watson dynamics are *R*_crit_ = 3.01, 2.91, 2.55, for Gaussian, Laplace and ExpSqrt dispersal, respectively.

There are two striking results concerning the DTDS-derived values of *R*_crit_. First, like the wave-width results, the critical radii are relatively insensitive to the dispersal model. Second, however, unlike the wave-width results, the critical radii are significantly different and smaller than those produced by the diffusion approximation. Barton and Turelli (2011) showed that the Schraiber et al. (2012) dynamics produced smaller *R*_crit_ values than the cubic model, even if fitness costs were purely based on reduced viability. Reduced fecundity, as assumed in the Caspari-Watson model, accelerates the local dynamics and hence allows much smaller introductions to initiate a traveling wave. Even with *p̂* = 0.35, introductions with *p*_0_ = 0.8 will succeed as long as the initial radius of release, denoted *R*_I_, satisfies *R*_I_ ≥ 2.5*σ* (or 3.0*σ*) with ExpSqrt (or Gaussian) dispersal.

## 5. Results: Predictions for 2013 Cairns releases

### 5.1. Diffusion-based predictions

One of our primary aims is to understand the robustness of the Barton and Turelli (2011) diffusion predictions. Rather than discuss generalities, we will focus on specific field releases. In early 2013, three localized releases were performed within the city of Cairns. Releases were made in three neighborhoods, Edgehill/Whitfield (EHW), Parramatta Park (PP), and Westcourt (WC). The release areas were roughly 0.97 km^2^ for EHW, 0.52 km^2^ for PP, and only 0.11 km^2^ for WC. Infection frequencies quickly rose above 0.8 within all three release areas, and each release area adjoined housing into which the *w*Mel infection might plausibly spread. What predictions emerge from the diffusion approximations?

Numerical predictions require estimates of *σ* and *p̂*. Russell et al. (2005) performed a mark-release-recapture experiment with *Ae. aegypti* using a release site abutting the 2013 EHW release area. The mean absolute distance of recaptures from the release point was about 78 m. The diffusion approximation assumes that dispersal is measured as the standard deviation of dispersal distance along any axis. If we assume that dispersal distance is roughly Gaussian distributed with mean 0 and standard deviation *σ* along each axis, the mean absolute dispersal distance is $\sigma \sqrt{\pi /2}$ or about 1.25*σ*. With this assumption, the estimate from Russell et al. (2005) implies *σ* ≈ 62 m/(generation)^1/2^. In general, however, release-recapture estimates tend to be systematically lower than those based on genetic data (see, for instance, Barton and Hewitt 1985, Fig. 3). Moreover, estimates of dispersal distance for *Ae. aegypti* are extremely variable in time and space. For instance, Harrington et al. (2005) found that repeated estimates of mean dispersal distance in the same village in Thailand ranged from about 40 m/(generation)^1/2^ to about 160 m/(generation)^1/2^. Our theoretical predictions concerning the consequences of dispersal are best interpreted as temporal averages, which are more likely to be accurately captured by indirect estimates of average dispersal such as wave width (or genetic data describing the decline of relatedness with distance). Given that direct estimates systematically underestimate average dispersal in nature, we use *σ* ≈ 100 m/(generation)^1/2^ as a plausible estimate for Cairns. We recognize, however, that dispersal is likely to vary significantly with local conditions.

Assuming *σ* ≈ 100 m/(generation)^1/2^, Eq. (6) implies that if spatial spread is observed, the wave width should be about 400 m. From Eq. (5), the corresponding wave speed is *c* = 100(½ – *p̂*) m per generation (m/gen). As argued in section 6.1 below, *p̂* is probably above 0.2. Thus, the maximum predicted speed is about 30 m/gen. However, if *p̂* is as high as 0.35, predicted speed falls to 15 m/gen. Assuming about 10 *Ae. aegypti* generations per year near Cairns, these crude estimates indicate that *w*Mel spread in *Ae. aegypti* is likely to be on the order of 150-300 m/year – two or three orders of magnitude slower than the spread of *w*Ri in California and eastern Australia *D. simulans* (100 km/year, Kriesner et al. 2013). Yet repeated estimates of dispersal distances for various *Drosophila* species suggest that natural dispersal distances are at most 5-10 times greater for *D. simulans* than for *Ae. aegypti* (*e.g*., Dobzhansky and Wright 1943; Powell et al. 1976; McInnis et al. 1982). The critical difference between the speeds associated with these exemplars of *Wolbachia* spread is unlikely to be dispersal, but more probably the bistability of *w*Mel dynamics in *Ae. aegypti* versus the monostability of *w*Ri dynamics (see Discussion section 8.3). Monostability allows relatively rare human-mediated, long-distance dispersal to greatly enhance spatial spread, as described, for instance, by “structured diffusion” models (Shigesada and Kawasaki 1997, Ch. 5).

Whether spatial spread occurs with bistability depends on the size of the release area, the initial frequency produced in the release area (*p*_0_), and *p̂*. From Fig. 3 of Barton and Turelli (2011) with *p*_0_ = 0.8, if *p̂* were as large as 0.35, the minimum radius of a circular release needed to produce an expanding wave would be on the order of *4σ*, implying a minimal release area of about 0.5 km^2^ (assuming *σ* ≈ 100 m/(generation)^1/2^). Replacing the cubic in (2) with the Schraiber et al. (2012) description of CI dynamics (3), Barton and Turelli (2011) showed that the minimal radius with *p̂* = 0.35 falls from about 4*σ* to about 2.8-3.5*σ*, with the value depending on whether *w*Mel-infected *Ae. aegypti* lose fitness primarily through fecundity (as the data of Hoffmann et al. 2014 suggest), which produces 2.8*σ*, or viability, which produces 3.5*σ*. The smaller values (from Schraiber et al. 2012) imply minimal release areas of about 0.25-0.38 km^2^ (the lower value assumes only fecundity effects). In contrast, if *p̂* were as small as 0.2, the minimum radius falls to about 2*σ* for both the cubic model and Schraiber et al. (2012) dynamics (with either fecundity or viability effects), corresponding to a minimal area of about 0.13 km^2^.

Table 1 summarizes our diffusion-based predictions. Note that according to these analyses, the releases at EHW and PP should certainly lead to spatial spread, but the WC release is close to minimal release area even if *p̂* is as small as 0.2. Next we address the robustness of these predictions to long-tailed dispersal and patchy spatial distributions.

### 5.2. DTDS-based predictions

Our robustness analyses of the wave-width predictions emerging from the cubic-diffusion model indicate that *σ* can be reliably estimated from observed widths of traveling waves of *Wolbachia* infections. In contrast, our wave-speed analyses suggest that given an estimate of *σ*, the predicted wave speed depends significantly on the shape of dispersal with plausible speeds that may be on the order of 20-30% below the cubic-diffusion prediction c=sh(12−p^).

Our final prediction concerns spatial spread from individual localized releases. As shown in Fig. 5, the critical release radius for spread depends on: 1) *p̂*, the unstable point; 2) *p*_0_, the initial infection frequency produced within the release areas; 3) the shape of the dispersal function; and 4) *σ*, dispersal distance. As dispersal becomes more long-tailed (moving from Gaussian to ExpSqrt), the critical radius of the initial introduction decreases. If we assume that *p̂* = 0.3 and *p*_0_ = 0.8, *R_c_*_rit_ is about 2.61*σ* if dispersal is Gaussian, but falls to about 2.51*σ* (or 2.16*σ*) if dispersal is Laplace (or ExpSqrt). Hence, for each of the three release areas in Cairns, we can ask what is the maximum *σ* consistent with our deterministic predictions for spatial spread. Given that spread occurs only if the release area exceeds $\pi R_{\text{crit}}^{2}\sigma^{2}$, for each release area, we can approximate an upper bound on σ consistent with spatial spread by

(10) $$\sigma <\sqrt{\left(\text{release area})/(\pi R_{\text{crit}}^{2}\right)}.$$

Table 2 presents these upper bounds on σ associated with the three 2013 release areas in central Cairns for a plausible range of *p̂*.

Given that very few empirical estimates of *σ* for *Aedes aegypti* exceed 100 m, these results suggest that spatial spread should certainly be observed for the Edge Hill/Whitfield and Parramatta Park releases. The prediction for Westcourt is more ambiguous. Note that from Table 1, our diffusion predictions with 0.2 ≤ *p̂* ≤ 0.35 indicated a minimum release area of 0.14 km^2^. This lower bound assumes *σ* = 100 m and *p̂* = 0.2. Thus the diffusion analyses suggested probable failure of the Westcourt release. In contrast, as shown in Fig. 5, our DTDS analyses indicate that the Westcourt release area may be near the lower limit for spread, with the outcome depending critically on the exact values of *σ* and *p̂*.

Empirically testing these predictions concerning minimal release areas is confounded by the fact that dynamics very close to the critical values for spread are expected to be slow. Assuming that *p̂* = 0.25, if the release area is 10% (5%) smaller than the critical value, the time for collapse is on the order of 15-20 (20-25) generations, roughly two years. Conversely, if the release area is only 10% (5%) larger that the critical area, the time scale for appreciable spatial spread is also on the order of 15-20 (20-25) generations. In contrast, release areas twice as large as necessary should produce appreciable spread in only 10-15 generations; whereas release areas only half as large as needed should essentially collapse in 10-15 generations. These calculations motivated our analyses presented below of “optimal” release sizes aimed at area-wide coverage within a few years.

## 6. Results: Data relevant to bistability and long-distance dispersal

### 6.1. Heuristic approximation for p̂ from Pyramid Estates data

Pyramid Estates (PE) was sampled for over two years after the releases stopped. The few capture sites were scattered over an area of houses that is on the order of 1 km^2^ with traps varying between about 100 m and 500 m from the nearest residences in our release area. For over two years, the *w*Mel frequency in PE remained persistently low, but non-zero with *p̄* ≈ 0.106 (*N* = 2689, averaged over space and time). (We found no evidence that infection frequency varied with distance from the release area). For instance, a sample of 43 *Ae. aegypti* from the week ending 9 January 2015 yielded an infection frequency of 0.07 [with 95% binomial confidence interval (0.01, 0.19)]. From Eq. (9), a long-term average of 0.105 implies *p̂* ≥ 0.21. The persistence of a low infection frequency for over two years clearly demonstrates regular immigration of infected individuals that has been unable to push the local PE population past its unstable point. The fitness data from Hoffmann et al. (2014) suggest that *p̂* for *w*Mel near Cairns is likely to be at least 0.2. This is corroborated by the transient dynamics described in Hoffmann et al. (2011) which also suggest that *p̂* is unlikely to be significantly above 0.3.

### 6.2. Long-tailed dispersal

Gordonvale and Yorkeys Knob are separated from other sizable populations of *Ae. aegypti* by kilometers. Yet, Hoffmann et al. (2014) found consistent low frequencies of uninfected individuals more than three years after *w*Mel reached near-fixation, despite no evidence for imperfect maternal transmission. Yorkeys Knob is less isolated than Gordonvale and shows a significantly higher frequency of uninfected individuals, about 6% versus 3%. Long-distance dispersal is the most plausible explanation for uninfected individuals in Gordonvale and Yorkeys Knob – and the persistence of rare infected individuals at Pyramid Estate.

## 7. Results: Near-optimal release strategies

We seek conditions under which releases of disease-suppressing Wolbachia transinfections achieve area-wide control of a disease such as dengue (cf. Ferguson et al. 2015) by transforming a significant fraction of the vector population, say 80%, in a relatively rapid period, say two to four years (on the order of 20-40 generations), while releasing as few Wolbachia-infected vectors as possible. We consider several questions associated with the optimizing the timing, spacing and intensity of releases. First, we contrast pulsed releases, over a time scale of very few vector generations, with prolonged low-intensity releases. Second, we consider optimizing the spacing and intensity of releases, as quantified by three parameters: a) local initial infection frequencies after releases, b) areas of local releases, and c) the spacing of releases. Third, given that optimization requires knowing parameters that can only be approximated, we consider the consequences of non-optimal releases.

### 7.1. Timing of releases: pulse versus gradual introduction

With bistable dynamics, the frequency of an infection (or allele) must be raised above a critical threshold, *p̂*, over a sufficiently large area to initiate spread. What is the most efficient way to establish an infection? At one extreme, the frequency could be raised essentially instantaneously to some *p*_0_(*x*); if *p*_0_ > *p̂* over a large enough region (cf. Fig. 5), the infection will spread. At the other extreme, there might be a gradual introduction, described by a local introduction rate *m*(*x*), sustained until deterministic spread is initiated. If this input is sufficiently high over a sufficiently large region, the infection will be locally established and spread. Between these extremes, releases might be sustained for a set period of many months or a few years. Supporting Information Appendix B investigates conditions for local establishment and wave initiation, providing analytical results for a single deme and for a point source of introduction in one dimension, and numerical results for two dimensions. We show that it is most efficient to raise infection frequency rapidly, in a brief pulse, rather than making gradual introductions. This accords with the intuition that it is most efficient to raise the frequency as quickly as possible above the threshold *p̂*: this maximizes the reproductive value of introduced individuals. The principle is simple; during gradual introductions, until local infection frequencies exceed *p̂*, the introduced infected individuals are systematically eliminated by deterministic selection that dominates the weaker (frequency-dependent) force of CI at low *Wolbachia* frequencies. Assuming that releases quickly drive the local infection frequency to a value *p*_0_ sufficient to initiate spatial spread, we ask how long it might take to cover a large area and what spatial patterns of release minimize the time to reach a desired coverage.

### 7.2. Spacing and intensity of releases

We start with idealized analyses, then discuss their relative robustness and the effects of environmental heterogeneity. Consider an area with a relatively uniform vector density. What is the optimal release strategy? The calculations in Appendix B show that for a given number of mosquitoes, the best strategy is to release a short pulse, *i.e*., to essentially instantly produce a local infection frequency sufficient to initiate a wave. Obviously there are practical constraints on numbers that can be released, as well as density-dependent effects, that limit the rate of local transformation. However, empirical results of Hoffmann et al. (2011) demonstrate that patches on the order of 1 km^2^ can be converted to relatively high *Wolbachia*-infection frequencies, on the order of 0.8, within two or three months. For simplicity, we focus on releasing *Wolbachia-*infected mosquitoes in circular areas of radius *R*_I_ that will form expanding waves. Because the expansion rate approaches zero as the release radius approaches the critical size threshold needed to produce an expanding wave, *R*_I_ must exceed this critical size. We assume that because of limitations associated with density regulation and constraints on numbers released, the highest initial frequency, *p*_0_, that can plausibly be achieved in each release area is *p*_max_ < 1. We consider laying out release areas in a uniform grid with spacing *D* between the centers of each release.

We envision expanding waves from each release. When the waves meet, the radius of each infected patch is *D*/2 and the fraction of the space occupied by *Wolbachia*-transformed mosquitoes is *π*/4 = 0.785 (i.e., *π*(*D*/2)^2^/*D*^2^), or roughly 80%. If the waves were instantly moving at the asymptotic speed *c*, they would meet in (*D*/2 – *R*_I_)/c time units. The actual time will be slower because the infection frequency must rise in the release area and the proper wave shape establish. Given that we can control *p*_0_ (≤ *p*_max_), *R*_I_, and *D*, we can ask: what values of these three parameters produce waves that meet in a minimum time for a fixed number of mosquitoes released – and what is that time? Alternatively, we can ask what is the minimum number of mosquitoes that must be released to produce advancing waves that meet within a fixed time? Given practical constraints on achieving specific values for *p*_0_, *R*_I_, and *D*, we then consider how sensitive our results are to these parameters and to model assumptions concerning dynamics and dispersal.

### 7.3. Empirically based approximations for area-wide coverage

Before addressing these questions with detailed dynamic models, we provide informative approximations from empirical results. From the data reported in Hoffmann et al. (2011) and Hoffmann et al. (2014), we know that releases of *w*Mel-infected *Aedes aegypti* can be used to stably transform areas with radius roughly *R*_I_ = 400 m. A *Wolbachia* frequency of about 80% within such release areas can be achieved in about 10 weeks (under three generations) by releasing weekly a number of adults on the order of 50-100% of the resident adult population (Hoffmann et al. 2011; Ritchie et al. 2013). Our theoretical analyses above and in Barton and Turelli (2011) suggest that rates of spatial spread are likely to be habitat dependent. But in relatively uniform habitats, comparable our release areas near Cairns with *σ* ≈ 100 m/(generation)^1/2^ and *p̂* ≈ 0.25-0.3, we expect wave speeds on the order of 10-20 m per month.

To understand the consequences of slow spatial spread, we initially consider dividing the target region into non-overlapping *D* × *D* squares. We will determine the value of *D* that achieves about 80% coverage over the desired period. Suppose that at the center of each square, we release *Wolbachia*-infected mosquitoes over a circle of radius *R*_I_. Assume that each release initiates a wave moving *c* meters per generation (roughly per month). If we want the expanding circles to hit the edges of the *D* × *D* squares within *T* generations, the wave front must move a distance *D*/2 – *R*_I_ in *T* generations. Hence, the distance between adjacent centers must be

(11) $$D=2(R_{I}+cT).$$

The fraction, *F*, of the target area that must be actively transformed to achieve *π*/4 coverage in *T g*enerations is *F* = *πR*_I_^2^/*D*^2^, where *D* is given by (11). Thus,

(12) $$F=\pi R_{I}^{2}/[4{\left(R_{I}+cT\right)}^{2}].$$

Table 3 shows how *F* depends on time (*T*, in generations), wave speed per generation (*c*), and the initial release radius (*R*_I_). The target times correspond roughly to one-to-four years. These approximations make sense only if the initial frequency in the release area is high enough that the asymptotic wave speed is reached within a few generations. They imply that for relatively homogeneous target areas consistent with steady spatial spread, roughly 80% can be covered in three or four years with initial releases of 0.5-1 km^2^ that cover about 10-30% of the target. Comparable results are obtained below from explicit dynamic models for wave initiation and spread.

**Fig. 6:**
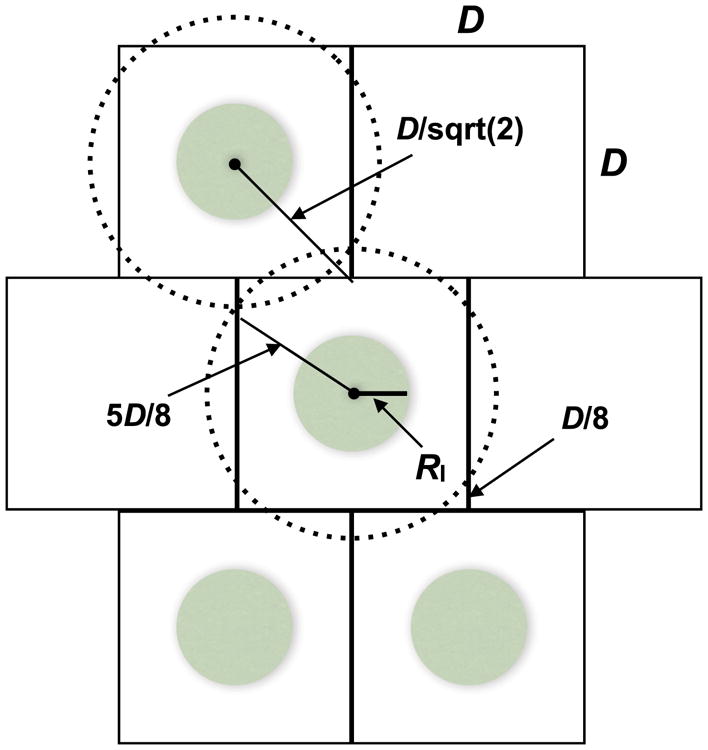
Optimal spacing. The green circles within the *D* × *D* squares represent release areas with radii *R*_I_. If the release areas were laid out on a regular grid, each expanding wave would have to travel to the corner of the enclosing square, a distance of (*D*/√2) – *R*_I_, to transform the entire target area. In contrast, by offsetting the release centers between adjacent rows, as illustrated, each wave must travel only (5*D*/8) – *R*_I_ for area-wide transformation.

To completely cover a region as quickly as possible, a regular grid of releases is not optimal. Fig. 6 shows how rows of releases with the centers offset between adjacent rows reduces the distance each wave must travel by *D*/√2 – 5*D*/8 ≈ 0.08*D*. (Note that with the release configuration shown in Fig. 6, when the radii of the expanding waves reach 5*D*/8, the entire target area has been transformed.) The empirical relevance of such idealized release spacings is considered in the Discussion.

### 7.4. Model-based approximations

Next, we reconsider the times to achieve roughly 80% coverage using explicit models for temporal and spatial dynamics. With explicit dynamics we can address various questions involving, for instance, optimal size and spacing of release areas and optimal initial frequencies in the release areas. Release areas have a major impact on subsequent dynamics. For releases near the minimal sizes required to initiate spread (cf. Fig. 5), dynamics will be extremely slow. In contrast, our calculations above assume that asymptotic wave speed is reached essentially instantaneously. Assuming Caspari-Watson dynamics with alternative dispersal models, we use the DTDS approximations (7) to describe optimal release strategies under different constraints.

#### 7.4.1. Optimal spacing and sizes of releases

For these calculations, we assume that releases occur in a fixed fraction, *ρ*, of the target area and that the initial *Wolbachia* frequency within the release areas is *p*_0_. To understand fully how mosquito releases translate into local infection frequencies, density regulation must be understood. Instead, we consider *ρ* and *p*_0_ as simple proxies for release effort. As above, we assume that release areas are circles of radius *R*_I_ set at the centers of *D* × *D* squares that cover the target area. Given *ρ*, the spacing *D* dictates the radii, *R*_I_, of the releases, with $R_{I}=D\sqrt{\rho /\pi }$. For fixed *ρ*, we seek the spacing *D* (or equivalently the release area) that minimizes the time until the waves meet (covering *π*/4 of the target area). The minimal time is denoted *T*_min_.

Assuming releases over 20% of the target area (*ρ* = 0.2) with initial infection frequencies, *p*_0_, of 0.6 or 0.8 in each release area, Table 4 presents optimal spacing for releases and the number of generations to reach 80% coverage for two plausible values of *p̂*. What seems most notable is that for these parameters, the optimal release radii are only about 30-45% larger than the minimum radii needed to initiate spatial spread. With “optimal” spacing, 80% coverage is predicted in about 1.25-3.5 years, assuming about 10 generations per year. The values of *T*_min_ are considerably smaller than those reported in Table 3, and the critical difference is that the release areas are considerably smaller. Table 3 assumes *σ* = 100 m, so the release sizes are fixed at *R*_I_ = 4 and 5.6. The shorter times in Table 4 are associated with the fact that in principle smaller releases will suffice to start waves that relatively quickly approach their asymptotic speed.

Table 4 shows that *T*_min_ depends only weakly on the shape of dispersal. As expected from our speed calculations, long-tailed dispersal leads to longer wait times. Two factors contribute to this, the differences in wave speed demonstrated in Fig. 3 and the differences in the optimal spacing. With longer dispersal tails, wave speed slows down, but the optimal spacing is closer (because smaller release radii suffice to initiate spread), and these effects partially cancel. In contrast, as *p̂* increases from 0.2 to 0.3, *T*_min_ increases by 70-80%, whereas the analytical prediction c=σsh(12−p^) and the numerical results in Fig. 3 indicate that wave speed should decrease by only about 50%, at most. The additional factor explaining the discrepancy is that larger releases are needed, producing larger spacing, *D*, so that the waves must travel farther to meet. Table 4 also predicts how *T*_min_ varies with the number of infected mosquitoes released, as measured by *p*_0_. As expected, the critical spacing, *D*, and the minimal time, *T*_min_, fall as initial frequencies rise. For instance, with Gaussian dispersal and *p̂* = 0.3, (*D*, *T*_min_) fall from (13.06, 17.60) with *p*_0_ = 0.6 to (10.74, 14.25) with *p*_0_ = 0.8 and to (9.36, 12.03) with *p*_0_ = 1.0. Overall, decreasing *p*_0_ from 0.8 to 0.6 leads to lengthening *T*_min_ by a factor of 1.20-1.25.

#### 7.4.2. Optimal distribution: release area, ρ, versus initial frequency, p_0_

Optimization depends on constraints. Above we assume that *ρ* and *p*_0_ have been chosen, then seek the optimal spacing (or equivalently the optimal sizes for the individual release areas), conditioned on *ρ*, the total area over which releases will occur. An alternative is to assume that available resources dictate the number of mosquitoes that can be released, then ask whether it is more efficient to produce a low initial frequency over a large area or a higher frequency over a smaller area. In general, we expect that achieving a frequency of 0.45 requires less than half the effort required to achieve 0.9 for at least two reasons. First, density-dependence is likely to produce diminishing returns from very intensive releases (Hancock et al. 2016); and second, very high frequencies can only be achieved with repeated releases, which are less efficient than more intense releases over shorter periods. Nevertheless, if we view that product *ρ p*_0_ as proportional to total release effort, it is instructive to ask for a fixed *ρ p*_0_ what *p*_0_ achieves 80% coverage as quickly as possible?

**Fig. 7:**
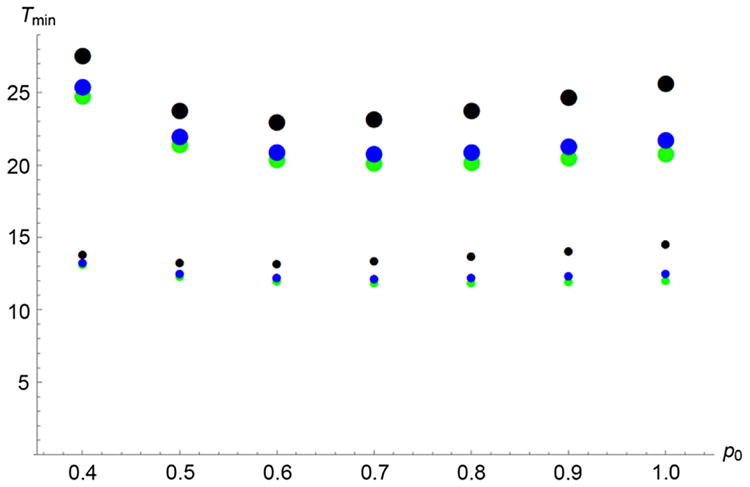
Time to reach about 80% (∼π/4) coverage as a function of initial frequency in the release area, *p*_0_. The calculations assume *ρ p*_0_ = 0.2, where *ρ* is the fraction of the target area over which releases occur. The small dots are produced with *p̂* = 0.2; the large dots with *p̂* = 0.3. Green points are for Gaussian dispersal, blue points for Laplace, and black for ExpSqrt.

Using all three dispersal models and *p̂* = 0.2 or 0.3, Fig. 7 plots the minimal time to achieve 80% cover as a function of *p*_0_ assuming *ρ p*_0_ = 0.2. The results indicate that releases producing initial frequencies between roughly 0.5 and 0.8 are essentially equivalent, with coverage times varying less than 10%. In contrast, the considerable additional effort required to produce *p*_0_ ≥ 0.9 yields slightly slower rather than faster coverage. Conversely, reaching only *p*_0_ = 0.04-0.5 requires significantly larger optimal release areas and yields slower coverage. For instance, with Laplace dispersal, *p̂* = 0.3, and *ρ p*_0_ = 0.2, *T*_min_ is achieved with *R*_I_ = 4.10 for *p*_0_ = 0.7 but *R*_I_ = 5.84 for *p*_0_ = 0.5, corresponding to roughly doubling the release areas. These results suggest that releases should aim for initial *Wolbachia* frequencies in the neighborhood of 60-80%.

#### 7.4.3. Robustness of coverage times to incomplete knowledge

Although one can propose optimal spacing and release areas for fixed *ρ* and *p*_0_, the optimal values are unlikely to be achieved in practice because they depend critically on two parameters, the local dispersal parameter *σ* and the value of the unstable equilibrium *p̂*, that will be known only approximately. Moreover, the geometry of field releases will be influenced by factors such as housing density and type, barriers to wave movement, and local community acceptance. Although the fraction of the target area in which releases are initially performed, *ρ*, is clearly under experimental control, as is the initial frequency in those release areas, *p*_0_, it is important to understand the robustness of the minimum times presented in Table 4 and Fig. 7 to alternative release areas, *R*_I_, which are measured in units of *σ*.

**Fig. 8:**
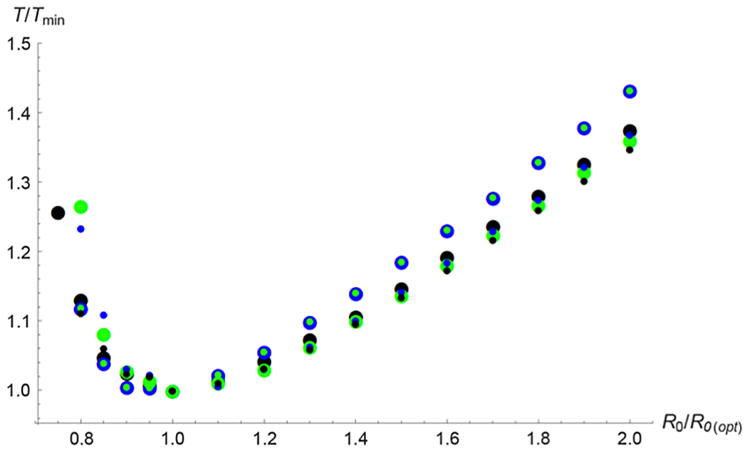
Time to reach about 80% (∼*π*/4) coverage, relative to the minimum time, as a function of release area. For each model, release areas are measured relative to the release area, *R*_I(opt)_, that produces *T*_min_ for that model. The DTDS calculations assume Caspari-Watson dynamics with *ρ* = 0.2 and *p*_0_ = 0.8 (i.e., releases produce an initial frequency of 0.8 over 20% of the target area). The small dots are produced with *p̂* = 0.2; the large dots with *p̂* = 0.3. Green points are for Gaussian dispersal, blue points for Laplace, and black for ExpSqrt.

Fig. 8 summarizes the results for all three dispersal models, assuming that we initially release over 20% of the target area (*ρ* = 0.2) and produce an initial infection frequency *p*_0_ = 0.8 relatively rapidly. As *R*_I_ departs from the optima given in Table 4, Fig. 8 shows how the time to achieve 80% coverage increases relative to _opt_, the minimal time achievable. As expected from Table 4, there is a fundamental asymmetry produced by the fact that the optimal *R*_I_ is typically only about 25-30% larger than the minimal release size needed to produce an expanding wave. Hence, undershooting the optimal release size by as little as 25% can lead to releases that collapse rather than expand. In contrast, for a realistic range of unstable points and all three models of dispersal, overshooting the optimal release area by 50% increases *T_π_*_/4_ by less than 20%. Even releases twice as large as optimal increase *T_π_*_/4_ by at most 43%. The clear implication is that one should use conservatively large estimates of *σ* and *p̂* to design releases that will produce near-optimal results with little possibility of collapse. The practical implications of Table 4 and Fig. 7 are discussed below.

## 8. Discussion

### 8.1 Robustness of the cubic-diffusion predictions for spatial spread

#### 8.1.1. Wave width

The point of estimating wave width is that it provides an average estimate – under natural field conditions – of the dispersal parameter *σ* that is central to predicting wave speed (see Eqs. 2.5 and 2.6). Using discrete-time, discrete-space (DTDS) approximations with alternative models of dispersal, we have tested the robustness of diffusion-based approximations for wave speed, wave width and the size of releases needed to initiate spatial spread. The most robust prediction concerns wave width (see Eq. 6 and Fig. 4). For a wide range of dispersal models and parameters, wave width is observed to be within about 10% of the analytical prediction, Eq. (6), produced by the cubic-diffusion approximation for bistable dynamics. This implies that estimates of the dispersal parameter *σ* can be obtained from data on the spatial pattern of infection frequencies after local releases. Unlike dispersal estimates obtained from short-term release-recapture experiments, estimates based on infection-frequency wave width average over seasons and are largely free from behavioral artifacts associated with inflated population densities or the effects of lab rearing, marking or handling.

#### 8.1.2. Wave speed

The cubic-diffusion model produces the wave-speed approximation Eq. (5): *c* = *σ*(½ – *p̂*) per generation, assuming complete cytoplasmic incompatibility (i.e., *s*_h_ = 1 in Eq. 3a or 4a). Our DTDS calculations show that this approximation remains accurate even for the rapid local dynamics produced by complete CI if dispersal is near-Gaussian (i.e., Gaussian or Laplace in Eq. 2.8) and the unstable point is below 0.4 (Fig. 3B). However, for long-tailed dispersal as described by the ExpSqrt model (see Eq. 8c and Fig. 2), spatial spread is slowed by 30-40% relative to the analytical prediction for 0.2 ≤ *p̂* ≤ 0.35. Hence, if *σ* is on the order of 100 m/(generation)^1/2^ and *p̂* is near 0.25, the predicted wave speed can drop from about 25 m/generation to about 15 m/generation. The result is that with about 10 *Ae. aegypti* generations per year, *w*Mel is expected to spread through natural populations of *Ae. aegypti* at a rate nearly three orders of magnitude slower than the 100 km/year rate at which *w*Ri spread through *D*. *simulans* populations in California and eastern Australia.

#### 8.1.3. Wave initiation

Finally, our DTDS calculations indicate that the cubic-diffusion approximations for the minimum radii of release areas from Barton and Turelli (2011) are likely to be significant overestimates, especially if fitness is reduced primarily through fecundity. Fig. 5 shows that the diffusion approximation may overestimate minimum release sizes by a factor of two for 0.2 ≤ *p̂* ≤ 0.35 (as noted in Section 4.3, most of this discrepancy is attributable to using a model that explicitly models Wolbachia dynamics, assuming that the cost of transinfections is mainly associated with a fecundity reduction). In general, for fixed *σ*, smaller releases will initiate spatial spread when dispersal is more long-tailed. With *σ* ≈ 100 m/(generation)^1/2^, releases that produce initial frequencies of 0.8 over about 0.13 km^2^ should suffice to initiate spatial spread, assuming that *p̂* ≤ 0.3. However, near this minimum, expansion (or collapse) is expected to be extremely slow, easily on the order of two years.

### 8.2. Predictions for 2013 Cairns releases

In 2013, the *w*Mel releases in the Edgehill/Whitfield (EHW) and Parramatta Park (PP) regions of Cairns quickly produced infection frequencies about 0.8 within the release areas (S. L. O'Neill, pers. comm.). Given that these sites are roughly 0.97 km^2^ (EHW) and 0.52 km^2^ (PP), we expect spatial spread of the infection from both release areas. Assuming *σ* ≈ 100 m/(generation)^1/2^ (corresponding to wave width on the order of 400 m), our analyses predict spread on the order of 10-25 m/generation, assuming *p̂* ≈ 0.25. In contrast, the Westcourt (WC) release encompassed only 0.11 km^2^, very close to the critical value that separates expected local establishment from collapse, assuming *p̂* ≈ 0.25 and 100 m/(generation)^1/2^ (see Table 2 for additional details). Given the slow rate of change expected near this threshold, considerable replication of such small releases would be required to convert our ambiguous prediction into a rigorous test. In contrast to the difficulty of testing our predictions concerning the minimum sizes of releases, our wave-speed and wave-width predictions can be easily compared to empirical data from urban field releases. The “Eliminate Dengue” project is currently preparing the data from the 2013 Cairns releases for publication.

### 8.3 Bistability for Wolbachia transinfections but probably not for natural infections

#### 8.3.1. Background

Early proposals by O'Neill and his collaborators (e.g., Sinkins et al. 1997) to transform natural populations with introduced *Wolbachia* were motivated at least in part by the belief that even fitness-decreasing infections might spread rapidly in nature, driven by the force of cytoplasmic incompatibility (Turelli and Hoffmann 1991, 1995). However, the rapid spatial spread of natural *Wolbachia* infections in *Drosophila* now seems dependent on net fitness advantages, previously unknown – and still not fully understood, that allow them to increase systematically in frequency even when they are so rare that cytoplasmic incompatibility provides no appreciable benefit (Fenton et al. 2011; Kriesner et al. 2013; Hamm et al. 2014). For *Wolbachia* infections that tend to increase when rare, occasional long-distance dispersal events can allow them to establish locally, spread and coalesce with other propagules, speeding their spatial spread far beyond what might be expected from more typical dispersal. Bistable dynamics, as produced by the appreciable fitness costs associated with *w*Mel-infected *Aedes aegypti* in Australia, restrict spatial spread to speeds set by average dispersal. Moreover, bistability sets a fundamental constraint on which transinfections might ever spread. S. L. O'Neill's “Eliminate Dengue” project (http://www.eliminatedengue.com/program) initially proposed introducing the life-shortening *Wolbachia*, *w*MelPop, into *Ae. aegypti* to greatly reduce the frequency of females old enough to transmit dengue virus. However, the fitness costs associated with *w*MelPop in *Ae. aegypti* produced an unstable infection frequency far above 0.5, precluding spatial spread (Barton 1979; Turelli 2010; Walker et al. 2011; Barton and Turelli 2011).

Turelli and Hoffmann (1991) proposed bistable dynamics to describe the northward spread of *Wolbachia* variant *w*Ri through California populations of *D. simulans*. The rationale for bistability was that the frequency-dependent advantage associated with CI seemed to be counteracted at low frequencies by two factors: imperfect maternal transmission, whereby a few percent of the ova produced by infected mothers were uninfected (Hoffmann et al. 1990; Turelli and Hoffmann 1995; Carrington et al. 2011); and reduced fecundity for infected females, with a 10-20% fecundity disadvantage observed in the lab (Hoffmann and Turelli 1988, Hoffmann et al. 1990; Nigro and Prout 1990) and a smaller, but statistically significant, fecundity disadvantage observed once in nature (Turelli and Hoffmann 1995).

The generality of bistable frequency dynamics for natural *Wolbachia* infections was brought into question by two infections found first in Australia that cause little (*w*Mel in *D. melanogaster*, Hoffmann 1988; Hoffmann et al. 1998) or no (*w*Au in *D. simulans*, Hoffmann et al. 1996) CI or other reproductive manipulation (Hoffmann and Turelli 1997). It was subsequently discovered that these *Wolbachia* nevertheless spread in nature. First noted was a turnover of *Wolbachia* variants among global populations of *D. melanogaster* (Riegler et al. 2005; Richardson et al. 2012), even though none of these variants cause appreciable CI when males are more than a few days old (Reynolds and Hoffmann 2002; Harcombe and Hoffmann 2004). Similarly, *Wolbachia* variant *w*Au, which does not cause CI in *D. simulans* (Hoffmann et al. 1996), was found spreading to intermediate frequencies through *D. simulans* populations in eastern Australia, despite imperfect maternal transmission (Kriesner et al. 2013). The spread of *w*Au was followed by the spread of *w*Ri through these same populations, beginning from three widely separated geographical locations (Kriesner et al. 2013). Although spread of bistable *Wolbachia* could in principle be initiated by chance fluctuations (Jansen et al. 2008), a net fitness advantage that counteracts imperfect transmission seems far more plausible (Hoffmann and Turelli 1997; Fenton et al. 2011; Hamm et al. 2014). The observed rate of spread for *w*Ri, approximately 100 km/yr., in both California and eastern Australia, is easy to understand only if long-distance, human-mediated dispersal can establish local infections that spread and coalesce (see Shigesada and Kawasaki 1997, Ch. 5). Such rapid expansion is implausible if local introductions must be sufficiently extensive to exceed initial area and frequency thresholds imposed by bistability (Lewis and Kareiva 1993; Soboleva et al. 2003; Alrock et al. 2011; Barton and Turelli 2011). With bistability, spatial spread is likely to be limited by the relatively slow processes of active insect dispersal. As demonstrated below, this indicates that the spread of transinfections with bistable dynamics in *Ae. aegypti* will be orders of magnitude slower than the 100 km/year observed for *w*Ri in California and Australia populations of *D. simulans*.

A net fitness benefit for natural *Wolbachia* infections helps explain the persistence and spread of *Wolbachia* variants, such as *w*Au and *w*Mel, that do not cause appreciable CI in their native *Drosophila* hosts. A net fitness benefit, so that the relative fitness of infected females, *F*, and their maternal transmission rate, 1 – *μ*, satisfy *F*(1 – *μ*) > 1, would also help explain the extraordinarily rapid human-mediated spatial spread of *w*Ri in both California and Australia. Mitochondrial data reported in Kriesner et al. (2013) suggest that *w*Ri spread northward in California shortly after it was introduced to southern California, rather than being stalled by a transverse mountain range, as might be expected with bistability (cf. Turelli and Hoffmann 1995). Several fitness advantages have been proposed to counteract imperfect transmission and possible fecundity disadvantages, including nutritional effects (Brownlie *et al*. 2009; Gill et al. 2014) and microbe protection (Hedges *et al*. 2008; Teixeira *et al*. 2008).

These arguments against bistability for natural *Wolbachia* infections may suggest that intrinsic fitness advantages, together with CI, could lead to rapid spread of disease-suppressing *Wolbachia* transinfections in nature from minimal introductions. The data we discuss in Section 6 argue strongly against this.

#### 8.3.2. New evidence for bistability of transinfections

Based on the theory in Barton and Turelli (2011) and the expectation that few mosquitoes would cross the highway, Hoffmann et al. (2011) predicted that “Unless fitness costs are essentially zero or there are unexpected fitness benefits, we do not expect the infection to spread further …” Four years later, *w*Mel has not become established in PE despite repeated immigration. An adaptation of Haldane's (1930) island model, Eq. (9), indicates a lower bound on the unstable equilibrium, *p̂*, of about 0.21. This local frequency threshold for population transformation appreciably slows the predicted rate of spatial spread, as indicated by Eq. (5).

Our new data and analyses bolster previous evidence for bistability. In Hoffmann et al. (2011), an informal quantitative analysis of the rising frequency of *w*Mel in response to several weekly releases indicated fitness costs on the order of 20%. However, the frequency data could not distinguish fitness costs associated with laboratory rearing from reduced fitness intrinsic to the *Wolbachia* transinfection. Two years later, Hoffmann et al. (2014) resampled these stably transformed populations and determined that the infected females produced about 20% fewer eggs under laboratory conditions, suggesting that *p̂* ≥ 0.2. As a consequence of bistability, the rate of spatial spread is limited by natural dispersal ability, with a maximum speed bounded above by *σ*/2 per generation, where *σ* is the dispersal parameter discussed below. In particular, bistability precludes very rapid spatial spread based on long-distance, human-mediated dispersal. Even when large numbers are transported by accident, the area transformed would be unlikely to exceed the minimum size needed to initiate spatial spread (Fig. 4).

The unstable equilibrium frequency, *p̂*, is a useful abstraction that captures key features of the complex frequency dynamics of *Wolbachia* transinfections. The true dynamics are multidimensional (Turelli 2010; Zheng et al. 2014) and depend on age-specific effects as well as ecological factors, such as intraspecific density-dependence (Hancock et al. 2011a,b; Hancock et al. 2016) and interaction with other insects and microbes (Fenton et al. 2011). However, a full description of this biology would involve many parameters that would have to be estimated in each locale. We doubt that these parameters could be estimated accurately enough for more realistic models to produce better predictions that our simple two-parameter approximations. Our idealized models of frequency dynamics produce field-testable predictions and empirically useful guidance for field releases.

### 8.4. Consequences of patchy population structure with bistable dynamics

We have assumed throughout a uniform population density and dispersal rate. In reality, habitat heterogeneity may slow – or stop – the spread of a wave. If increase is expected from low frequencies, then a few long-range migrants can take the infection beyond a local barrier. We expect this has happened repeatedly with the observed spread of *w*Au and *w*Ri in *Drosophila simulans* (cf Coyne et al. 1982; Coyne et al. 1987; Kriesner et al. 2013). Similarly, many episodes of successful long-distance dispersal and local establishment must underlie the global spread *of Aedes aegypti* out of Africa (Brown et al. 2011). However, bistability, as expected for the *w*Mel infection in *Ae. aegypti*, implies that infection spread can be stopped indefinitely, as seems to be the case with Pyramid Estate/Gordonvale near Cairns. Barton and Turelli (2011, Eq. 20) gave a simple result that shows how a gradient in population density alters wave speed: regardless of the detailed dynamics, a gradient in log density will slow (or accelerate) a travelling wave by *σ*^2^*d*(log(*ρ*(*x*))/*dx*, where *ρ*(*x*) denotes the population density at *x*. However, such a gradient must be sustained over a sufficient distance. Local heterogeneities, such as those due to the spacing between discrete demes (e.g., individual households harboring *Ae. aegypti*), have a negligible effect if they are over a shorter scale than the width of the wave (Barton 1979, p. 357).

In contrast, when the wave encounters a significant barrier, such as the highway separating Pyramid Estate from Gordonvale, we can understand wave stopping either in terms of sharp breaks in density, as considered in Fig. 6 of Barton and Turelli (2011), or in terms of migration from an infected population into an uninfected population. The latter produces a lower bound on immigration rate needed to “flip” the uninfected population past the unstable point, as discussed above Eq. 1. Because large tropical cities that are the targets of control efforts for arboviruses such as dengue and Zika are filled with significant dispersal barriers, we have not considered release schemes more elaborate than regularly spaced, equal-sized release foci. Nevertheless, we hope these abstractions accurately indicate the potential for area-wide control with plausible effort over a span of a few years.

### 8.5. Practical guidelines for field releases

When the “Eliminate Dengue” program initially obtained Gates Foundation “Grand Challenges” funding in 2006, the extraordinarily rapid spread of *w*Ri through California populations of *D. simulans* provided a plausible paradigm supporting the conjecture that natural *Ae. aegypti* populations could be rapidly transformed with disease-suppressing *Wolbachia*. The *D. simulans* paradigm also suggested that very few local introductions could lead to area-wide transformation within a few years for large metropolitan areas with relatively continuous *Ae. aegypti* habitat. Unfortunately, this rapid-spread paradigm, which remains demonstrably true for natural *Wolbachia* infections (Kriesner et al. 2013), now seems clearly inapplicable to *Wolbachia* transinfections that significantly reduce the fitness of their *Ae. aegypti* hosts. More plausible rates of spatial spread seem to be at most 0.25 km per year, and even those slow rates are expected only in near-continuous habitats. From our analysis of the Pyramid Estate data, it seems that barriers on the order of 100-200 m, such as highways, will suffice to halt spread. Hence, it is reasonable to ask whether spatial spread can play a significant role in achieving area-wide coverage over a time scale of a few years.

A central question is whether real urban/suburban landscapes provide enough nearly-continuous habitat to apply our optimal – or near-optimal – release designs, involving a series of releases set out in grids. We have showcased an empirical example in which *w*Mel has apparently not been able to cross a highway. We do not yet know enough to characterize a priori the barriers that will halt *w*Mel spread. What is clear is that area-wide control over just a few years will require many release areas. We can offer simple guidance based on our mathematical results and the population biology of vector-borne disease transmission. Given that spatial spread will preferentially occur from high-density areas to low-density areas, a guiding principle is that releases should initially occur in areas that support the highest *Ae. aegypti* densities. Because disease transmission is proportional to vector density, these areas are the natural targets for initial control efforts.

Our calculations provide more detailed guidance concerning the size of individual releases, their spacing, and the initial infection frequencies that should be achieved. Fig. 7 shows that for a wide range of parameters, releases need not produce initial frequencies above 0.6. Indeed, the effort to achieve much higher initial frequencies may produce slightly slower area-wide coverage, if a fixed fraction of the local mosquito population is initially replaced. As demonstrated by Fig. 7, overshooting optimal release areas even by a factor of two should increase the time to produce large-scale coverage by at most 50%. In contrast, Table 2 shows that “optimal” release areas are often only twice as large as the minimal release areas needed to initiate spread (corresponding to *R*_I_/*R*_crit_ = √2 in Table 2). Thus, release areas should be based on conservatively large estimates of *σ* and *p̂*. Assuming *σ* ≤ 120 m/gen^1/2^ and *p̂* ≤ 0.3, individual releases on the order of 1 km^2^, producing initial frequencies of 60-80%, should generally suffice to guarantee local spread, assuming that the surrounding habitat has population densities comparable to or lower than the release area. If the habitat is sufficiently homogeneous, covering only about a third of the target area with such releases should produce about 80% coverage in less than three years.

All of our guidelines are predicated on *p̂* ≤ 0.35. The lower the unstable point the better. But if there is any significant cost of *Wolbachia* transinfections, so that *p̂* ≥ 0.1, wave speed is likely to bounded above by *σ*/2. Although spatial spread of low-*p̂* variants is unlikely to be significantly aided by occasional long-distance dispersal, the spread of such variants is far less likely to be stopped by minor barriers to dispersal. As shown in Fig. 6 of Barton and Turelli (2011), step-increases in population density of just over two-fold will stop the spatial spread of a transinfection that produces *p̂* = 0.25; whereas an increase greater than five-fold is needed to stop a variant with *p̂* = 0.1.

Given that only two *Wolbachia* transfections of *Ae. aegypti* have been released in nature in population transformation efforts, we don't know whether there are *Wolbachia* variants that can provide effective virus-blocking and produce low fitness costs. In preliminary analyses, high *Wolbachia* titer is associated with better virus blocking and also lower fitness of infected hosts (Walker et al. 2011; Martinez et al. 2015). Among *Wolbachia* found in *Drosophila* species and transferred into *D. simulans*, the relationships between titer and measures of fitness loss and virus protection are both highly significant; but they explain only about half of the variation observed in each trait. Hence, it seems likely that further exploration of *Wolbachia* variation in nature could uncover high-protection, low-fitness-cost variants.

Despite the fact that the *w*Mel variant currently being released will spread very slowly and may be relatively easily stopped by barriers to dispersal, it still offers significant benefits over disease-control strategies like insecticide application and sterile-male release (or release of CI-causing males) that require continual applications to suppress local vector populations (McGraw and O'Neill 2013). As shown by Hoffmann et al. (2014), transformations of isolated populations with *Wolbachia* remain stable. Similarly, for sufficiently large local releases, we expect local *Wolbachia* introductions to at least persist and probably slowly expand as long as the surrounding areas do not harbor significantly higher *Ae. aegypti* densities. Even if half of a large area has to be actively transformed to achieve area-wide control, this will only have to be done once. We do not know how long-lasting dengue-blocking by *w*Mel or other transinfections will be, but the comparative evidence from natural *Wolbachia* infections suggests that it should persist for at least a decade or more (Bull and Turelli 2013), a time-scale over which effective vaccines may well become available (Screaton et al. 2015).

### 8.6. Final comment: reversibility versus re-transformation

Population transformation carries a potential risk of unintended consequences (Bull and Turelli 2013). For instance, a *Wolbachia* strain that inhibits the transmission of one disease may in principle enhance the transmission of another (cf. Martinez et al. 2014). Hence, it is interesting to ask whether an introduced *Wolbachia* can be “recalled”, returning the population to its initial uninfected state. In principle, this could be done by swamping the population with uninfected individuals so that the infection frequency falls below *p̂*. However, given the tendency of variants with *p̂* < 0.5 to spread spatially, this swamping strategy seems implausible outside of relatively small, isolated populations. It seems more plausible to re-transform populations with a more desirable *Wolbachia* variant that shows unidirectional incompatibility with the first. For example, when *Wolbachia w*Mel is introduced from *D. melanogaster* into *D. simulans*, which is naturally infected by *w*Ri, the *w*Mel-infected females are incompatible with *w*Ri infected males, whereas *w*Ri females are protected from the incompatibility that *w*Mel induces against uninfected females (Poinsot et al. 1998). Thus if a population has been transformed with *w*Mel, it could in principle be transformed again by introducing *w*Ri. The hit-and-miss process of identifying *Wolbachia* strains in nature with the desired properties is likely to be greatly accelerated as we begin to understand the loci within *Wolbachia* that cause CI (Beckmann and Fallon 2013; LePage et al. 2017; Beckmann et al. 2017) and virus inhibition.

## Supplementary Material

1 **S1 File. Appendix A.** Effect of dispersal pattern and random fluctuations on travelling waves.
**S2 File. Appendix B.** Establishing a wave.

2

3

## Figures and Tables

**Table 1:** Diffusion-based predictions for spatial spread

Unstable point	Speed (m/gen)	Width (m)	Minimum Release Area (km^2^)
*p̂* = 0.2	30	400	0.13
*p̂* = 0.35	15	400	0.25-0.38^a^ (0.5)

These predictions assume *σ* ≈ 100 m, *p*_0_ = 0.8, and 0.2 ≤ *p̂* ≤ 0.35. They are based on Eq. 5, Eq. 6 and Fig. 3 of Barton and Turelli (2011).

a The smaller prediction (0.25) is derived with Schraiber et al. (2012) CI dynamics (3) assuming that *w*Mel reduces only the fecundity of *Ae. aegypti* (i.e., *s*_r_ = *s*_f_ in 3b), the larger result (0.38) assumes that *w*Mel reduces only viability. The cubic model produces the still larger value (0.5).

**Table 2:** Predicted maximum *σ*(in meters) consistent with spatial spread

Location	*p̂*	Gaussian	Laplace	ExpSqrt
Westcourt (0.11 km^2^)	0.2	92	95	115
	0.25	81	84	101
	0.3	72	74	87
	0.35	62	64	73
Parramatta Park (0.52 km^2^)	0.35	135	140	160
Edge Hill/Whitfield (0.97 km^2^)	0.35	184	191	218

These prediction, based on inequality (10), assume that assuming that *p*_0_ = 0.8 and 0.2 ≤ *p̂* ≤ 0.35.

**Table 3:** Fraction of the target area that must be actively transformed to produce about 80% (π/4) coverage in *T* generations

*R*_I_ (area)	40 generations	30 generations	20 generations	10 generations
400 m (0.5 km^2^)	0.09-0.20	0.13-0.26	0.20-0.35	0.35-0.50
560 m (0.99 km^2^)	0.13-0.27	0.18-0.33	0.27-0.43	0.43-0.57

These calculations assume wave speed *c* = 10-20 m/generation starting from initial releases in circles of radius *R*_I_ that produce local infection frequencies *p*_0_ near 1.

**Table 4:** Optimal spacing of releases

*p*_0_ = 0.6	*p̂* = 0.2				*p̂* = 0.3			

Dispersal	*D*	*R_I_*	*R*_crit_	*T*_min_	*D*	*R*_I_	*R*_crit_	*T*_min_
Gaussian	13.06	3.30	2.48	17.60	16.26	4.10	3.19	30.22
Laplace	12.21	3.08	2.35	17.95	16.22	4.09	3.06	31.13
ExpSqrt	11.30	2.85	1.99	19.54	15.11	3.81	2.64	34.87

*p*_0_ = 0.8	*p̂* = 0.2				*p̂* = 0.3			

Dispersal	*D*	*R*_I_	*R*_crit_	*T*_min_	*D*	*R*_I_	*R*_crit_	*T*_min_

Gaussian	11.26	2.86	2.05	14.17	13.43	3.39	2.61	24.26
Laplace	10.25	2.60	1.97	14.57	13.39	3.38	2.52	25.09
ExpSqrt	9.34	2.36	1.62	16.25	12.18	3.07	2.16	28.59

All distances are measured in units of *σ*. Assuming releases over 20% of the target area (i.e., *ρ* = 0.2 and *p*_0_ = 0.6 or 0.8), we compare the spacing, *D* (distance between adjacent release centers), that produces the shortest time (in generations), *T*_min_, required to reach 80% coverage as a function of *p*_0_, initial infection frequency in release areas, *p̂* and dispersal shape. The initial radius of these optimal releases, $R_{I}=D\sqrt{\rho /\pi }$, is compared to the minimum radius, R_crit_, required to initiate an expanding wave for the specified *p*_0_ and *p̂*.
