# Supplementary material for: Deploying dengue-suppressing Wolbachia: robust models predict slow but effective spatial spread in Aedes aegypti
Source: Theor Popul Biol. Author manuscript; Available in PMC 2018 Jun 1. (PMC5476474; doi:10.1016/j.tpb.2017.03.003)
Supplement: 2 [file NIHMS868121-supplement-2.pdf]

Appendix A: Effect of dispersal pattern and random fluctuations on travelling waves

Ultimately, populations consist of reproducing individuals. In continuous space, they may be approximated by determinsitic integro-difference equations (IDE; Wang et al. 2002) in which allele frequencies or species density is taken to be cointinuous through space:

$$p_t[x] = \int \phi[x, y]\, g[p_{t-1}[y]]\, dy \qquad (1)$$

where $\phi[x, y]$ is the probability of moving from y to x . If change is sufficiently slow, and dispersal local, then this may be further approximated by a spatial diffusion, continuous in time, t, as well as space, x:

$$\partial_t p = \frac{\sigma^2}{2} \frac{\partial^2 p}{\partial x^2} + f[p] \qquad (2)$$

where $f[p] = g[p] - p$, and $\sigma^2 = \int \phi[\epsilon]\, \epsilon^2\, d\epsilon$ is the variance of dispersal distance. In the following numerical examples, we divide the habitat into discrete demes, and simulate a discrete stepping-stone model. However, we choose a large enough dispersal range that the deme spacing has negligible effect.

Under the diffusion approximation, the direction of movement is determined by whether the net rate of increase, $\int_0^1 f[p]\, dp$, is positive or negative. This simple result carries over to IDE models, and is independent of the form of dispersal, ϕ, provided that dispersal is symmetric. In stochastic models, the extent of spread (as measured by $\int p\, dx$, say) varies randomly, but the expected rate of spread still follows the same rule.

There is a qualitative distinction between two kinds of wave: *pushed* versus *pulled* (Stokes 1976). If $f[p] \geq p f'[0]$ for some $0 < p < 1$, then there will be a travelling wave that is pushed by increase within its bulk, and so which is insensitive to long-range dispersal, or to random fluctuations. With random sampling drift or demographic stochasticity, the variance in position increases by $\sim \frac{1}{N}$ in one dimension, where population density is N. In contrast, if $f < p f'[0]$ everywhere, then the wave is pulled at a rate determined by the individuals at the advancing front; individuals behind the wave almost all descend from this small fraction (Brunet et al. 2006). Now, the spread of the wave is sensitive to the form of long-range dispersal, and to the population density. Provided that the dispersal distribution is bounded by an exponential, the wave will settle to a steady shape, with a speed that depends only on the growth rate from low density; random fluctuations will slow it by an amount that depends logarithmically on density (Brunet et al. 2006). If the rate of long-range dispersal is faster than exponential, then the wave will accelerate, though again, random fluctuation will limit this acceleration, and cause the wave to fragment.

Effect of dispersal distribution on wave speed: deterministic models

We illustrate these points using the simple cubic model:

$$f[p] = \frac{s}{2} pq\,(p - q + \alpha) = spq\,(p - \hat{p}) \qquad (3)$$

where $p + q = 1$. Note that the model only makes sense for sufficiently small s: it can be taken as a weak-selection approximation to a variety of more detailed models (Barton and Turelli, 2011). However, it is accurate even for quite large s, and so we take it as a surrogate for a much wider class of models.

Because we will be considering the case $\hat{p} < 0$, it is more natural to use the parameter α in this section. Without loss of generality, we assume $\hat{p} < \frac{1}{2}$, or $\alpha = 1 - 2\hat{p} > 0$. If $\hat{p} > 0$, $\alpha < 1$, there is an unstable

equilibrium, whereas if $\hat{p} < 0$, $\alpha > 1$, the allele can increase from low frequency. ($\hat{p} = 0$ represents selection on a strictly recessive allele). If $\alpha < 3$ or $\hat{p} > -1$, then $f[p] \geq p f'[0]$ for some p, and so a pushed wave solution exists. However, a pulled solution also exists, with speed $c = \sigma\alpha\sqrt{s/4}$. The transition between a pulled and pushed wave occurs when the speeds of the two solutions intersect, at $\alpha = 2$, or $\hat{p} = -\frac{1}{2}$. For larger α, the deterministic solution is "pulled", with speed $c = \sigma\sqrt{s(\alpha-1)} = \sigma\sqrt{2\,s(-\hat{p})}$ (Fisher 1937).

Figure 1 shows how the wave speed increases with α, measured relative to the prediction for a pushed wave, $\alpha\sigma\sqrt{s/4}$ (horizontal line). For Gaussian dispersal, the speed is very close to the diffusion approximation for α<2, and close to the prediction for a pulled wave, c = $\sigma\sqrt{s(\alpha-1)}$, for $\alpha > 2$ (*see below*). A reflected exponential ("Laplace") distribution, which has substantially fatter tails, is also close to the diffusion approximation for a pulled wave (blue dots to right), but is slightly slower than predicted for a pushed wave for α<1. This is because genes that move a long way will be at a selective disadvantage, and so will be lost: the wave speed depends on the shape of the bulk of the dispersal kernel, which for a given variance, is narrower than a Gaussian (Fig. 2). The exponential square root distribution has even fatter tails, and shows a correspondingly slower speed for α<1. For α≥2, this fat-tailed distribution gives a higher speed, but still settles to a travelling wave with constant speed. This is at first puzzling, since distributions with fatter than exponential tails are predicted to give an accelerating wave. However, numerical calculations were made with a dispersal distribution truncated at ± 20 demes, or ±10 standard deviations (black dots). If this truncation is increased to 50 or 100 demes (purple and red dots), this makes little difference to the wave speed for $\alpha < 1$ (speeds are indistinguishable for α<1), but substantially increases the speed for $\alpha > 1$, as predicted. However, the speed is only increased by ~ 20% even when individuals can disperse out to 100 demes, or ± 50σ. Moreover, even for the exponential square root distribution, only an extremely small fraction of the distribution is excluded by truncation at 25σ. Arguably, it would be unrealistic to calculate for yet longer tails, since real ranges are finite, and since over such large distances, stochastic effects will dominate even in very large populations, as we discuss below.

Figure 1. Wave speed, c, relative to that expected for a pushed wave, $\alpha\sigma\sqrt{s/4}$, is plotted against $\alpha = 1 - 2\hat{p}$. The horizontal line at 1 is the expectation for a pushed wave, whilst the downward curve at the right is the ratio expected for a pulled wave, whose speed is c=$\sigma\sqrt{s(\alpha-1)}$. Dots show numerical calculations for three forms of dispersal: Gaussian (green), Laplace (blue), and exponential square root (black); the dispersal distribution is truncated at ±20 demes, and the standard deviation adjusted to equal σ=2. For the exponential square root, results are also shown for truncation at ±50 demes (purple) and ±100 demes (red). The selection coefficient is adjusted so that the maximum rate of change is always 0.025; this is equivalent to constant directional selection of $s = 20\,\%$. With these parameters, the deme spacing has negligible effect.

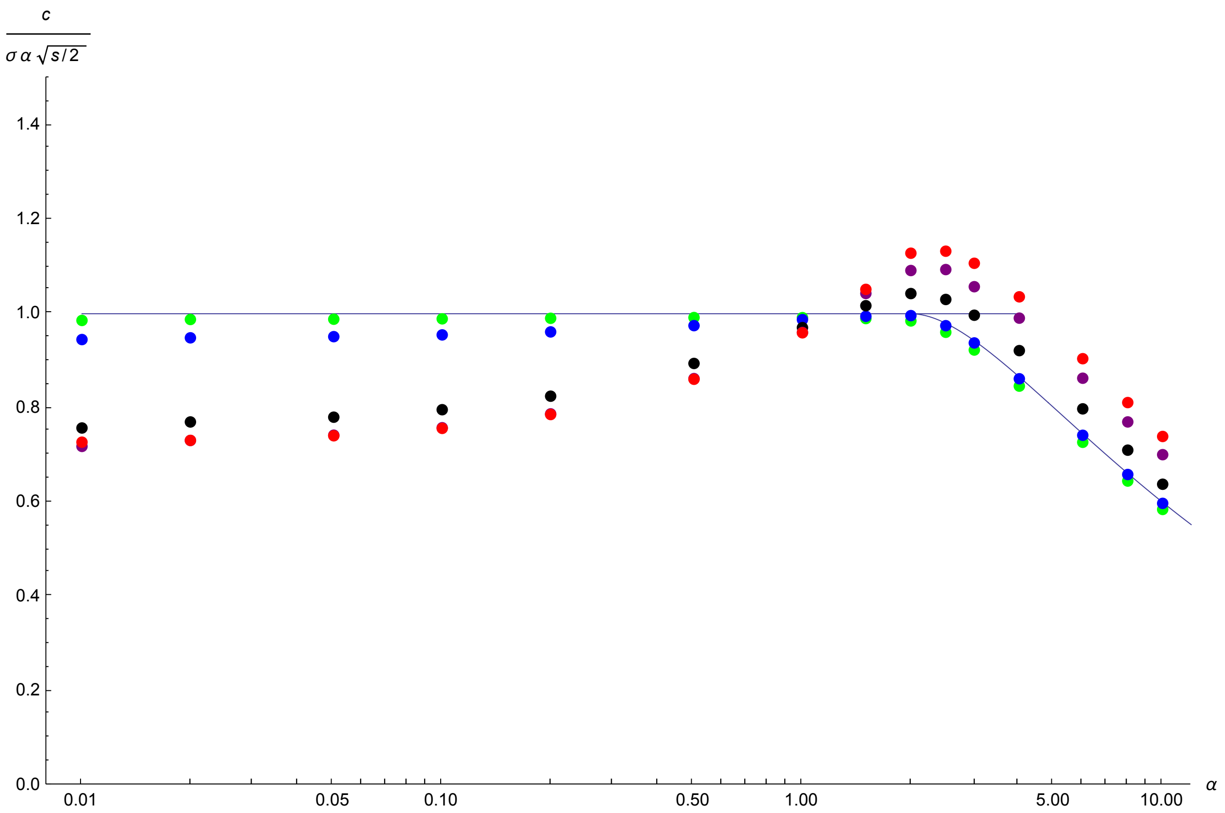

Table 1. The mass and the fraction of the total variance that is excluded by truncating distributions at 10σ (Fig. 1, green, blue, black), 25σ (Fig. 1, purple) and 50σ (Fig. 1, red).

		Gaussian	Laplace	ExpSqrt
10 σ	mass	1.52397×10^{-23}	7.21354×10^{-7}	0.000326548
	variance	1.55416×10^{-21}	0.0000830583	0.0513746
25 σ	mass	6.11339×10^{-138}	4.41948×10^{-16}	1.14081×10^{-6}
	variance	3.83308×10^{-135}	2.92285×10^{-13}	0.000934673
50 σ	mass	0.	1.95318×10^{-31}	1.67283×10^{-9}
	variance	0.	5.02302×10^{-28}	5.03447×10^{-6}

Wave speed with infinite variance

In Fig. 1, we compared dispersal distributions that were scaled to have the same variance. This approach fails for distributions such as the Cauchy, that fall away so slowly that they have infinite variance. However, we can find the Cauchy distribution that yields the same wave speed as a Gaussian with (say) $\sigma = 2$ (Fig. 3). On an infinite range, a Cauchy distribution leads to a wave with ever-increasing speed. However, the Cauchy distribution must necessarily be truncated for numerical calculations, and for any given truncation point, the wave does settle to a steady speed, which can be matched to that of a Gaussian. Figure 3 plots the standard deviation of the truncated Cauchy against $\alpha = 1 - 2\hat{p}$, for truncation at 10σ … 100σ (black … red dots). For pushed waves ($\alpha < 2$), fat tails slow down the wave, because long-distance migrants are lost, and so the truncated Cauchy must have a higher variance to maintain the same speed ($\alpha < 2$, left of Fig. 3). For a pulled wave, however, a truncated Cauchy with a very small variance can maintain high speed, provided that it is truncated sufficiently far out. In the most extreme case shown here (truncation at 100σ, α=4; bottom right of Fig. 3), 0nly a

fraction $7.9*10^{-8}$ disperse away from their native deme, contributing variance ~10^{-5}, and yet this maintains a wave with the same speed as Gaussian dispersal with standard deviation σ=2. (Note that for these extreme cases, the choice of deme spacing does affect the results, though not qualitatively).

Figure 2. The standard deviation, σ^*, of the truncated Cauchy distribution that gives the same speed as a Gaussian with σ=2 (horizontal line); this is plotted against α. The distribution is truncated at 10σ, 25σ, 50σ, 75σ, 100σ (black, blue, brown, purple, red).

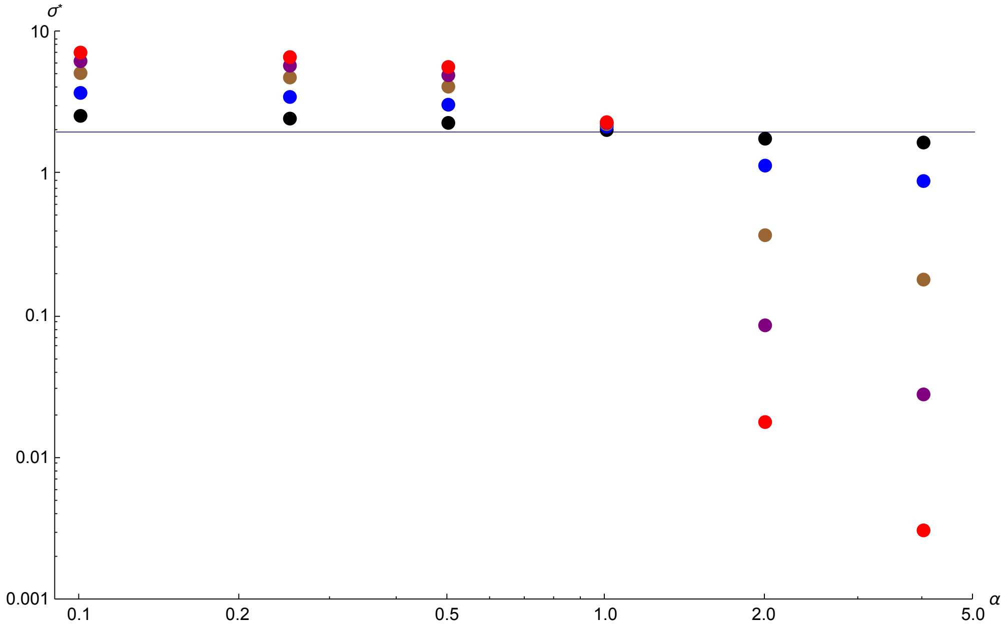

Random fluctuations

With long-tailed dispersal, deterministic models are misleading, because the numbers of long-range migrants will be small even in very dense populations. Indeed, even with Gaussian dispersal, finite population size has an appreciable effect on the speed of a pulled wave, because the dynamics depend on regions where alleles or individuals are very rare. Here, we simulate a finite population on an infinite range. The population is represented as a list of the numbers of the focal allele in each deme; all demes to the left are assumed to be fixed, and all to the right are assumed to be at zero. In each generation, and in each deme, there is selection followed by random sampling of 2N genes, following the Wright-Fisher model. Dispersal is implemented as follows. For each of the n_p demes that are currently polymorphic, the 2N genes are drawn from a parent population according to the dispersal function; the continuous dispersal distribution is rounded to the nearest integer. (For σ=2, as used here, this causes negligible error). These genes can be from arbitrarily far away. The same procedure is followed for 1000 demes to the left of the current set, and 1000 demes to the right. Finally, the list is trimmed to remove fixed demes on the left and on the right (but tracking the location of the leftmost polymorphic deme, which will tend to move to the right as the wave advances. This scheme is extremely efficient, since the random distances moved can be taken as a single draw of 2Nn_p random numbers from the dispersal distribution. The only approximations are that individuals live on a discrete grid, and that only ±1000 demes are followed on either side.

Table 2 summarises the effects of finite population size. For a pushed wave (α=0.5), random drift has little effect, unless deme size is very small ($2N = 5$). For a pulled wave, there is a substantial slowing, even for demes of $2N = 1000$ individuals. Note that this stochastic effect means that long range disper-

sal no longer increases the speed of advance, even for the exponential square root. There is no sign that rare propagules move far ahead of the initial wave, producing the kind of stochastic advance that is expected for very fat-tailed distribution. The key conclusion is that random fluctuations have very little effect on the speed of "pushed" waves. The slowing effect is greater when waves are "pulled", and there is long-range dispersal. However, even then the effect is not as dramatic as suggested by the asymptotic theory.

Table 2. The wave speed in a finite population. This is estimated as the mean speed over 2000 generations. The last row shows the results for the deterministic model (Fig. 1) with $k_{max} = 20$ for the Gaussian and Laplace distributions, and $k_m = 200$ for the exponential square root.

$\alpha = 0.5$	$2N$	Gaussian	Laplace	ExpSqrt	$\alpha = 4$	Gaussian	Laplace	ExpSqrt
	10	0.263	0.274	0.267		0.533	0.540	0.546
	100	0.259	0.257	0.243		0.697	0.672	0.705
	1000					0.716	0.704	0.746
	∞	0.255	0.250	0.230		0.751	0.765	0.920

References not cited in the main text.

Brunet E, Derrida B, Mueller AH, Munier S (2006) Phenomenological theory giving the full statistics of the position of fluctuating pulled fronts. Phys. Rev. E 73: 056126.
Fisher, RA (1937) The wave of advance of advantageous genes. Annals of Eugenics 7: 355-369.
Stokes AN (1976) On two types of moving front in quasilinear diffusion. Mathematical Biosciences 31: 307-315.
