# Supplementary material for: Deploying dengue-suppressing Wolbachia: robust models predict slow but effective spatial spread in Aedes aegypti
Source: Theor Popul Biol. Author manuscript; Available in PMC 2018 Jun 1. (PMC5476474; doi:10.1016/j.tpb.2017.03.003)

Appendix B: Establishing a wave

In a bistable system, a wave can be established if the initial distribution is above some threshold. Alternatively, one might continuously release within some region: again, there will be some threshold required for establishment. If the intensity of the source is above this threshold, then the wave can be established even if the source is turned off at a finite time. A key comparison is the total number that need to be introduced to establish the wave, or (more simply) how long the source needs to continue to send out the same number as would be needed with an initial brief pulse. Our intuition is that an initial pulse would always be more efficient, since any release in regions below the threshold will decay over time: the key is to raise a sufficiently large region above the threshold so that it can contribute to further increase.

We represent an influx by a term $mf[x]\,q$, which represents migration from a deme fixed for $p = 1$: in each generation, a (scaled) fraction $mf(X)$ is replaced by migrants. This ensures that allele frequencies do not rise above 1. If one follows population density, scaled relative to carrying capacity, and with an Allee effect that causes bistability, then one would add a term $\lambda f[x]$, and density could rise above carrying capacity. Here we assume a source $\lambda f(X) q$, corresponding to replacement of a fraction of the population. This is appropriate if population size is regulated to a constant value after the release.

Island model

Take the simple case of a single population, with an influx that replaces a fraction m of the population every generation. Let $T = (s_h/2)\,t$, $M = \frac{2m}{s_h}$; $\alpha = 1 - 2\hat{p}$:

$$\frac{\partial p}{\partial T} = pq\,(2p - 1 + \alpha) + Mq \tag{1}$$

Then, it is easy to show that the critical migration rate is $M^{*} = (1-\alpha)^2/16$. How much higher does M need to be if sustained for a finite time, T_0? Integrating Eq. B1, starting from zero allele freequency:

$$T = \frac{(3+\alpha)}{\sqrt{M - M^{*}}\,(1 + M^{*} + \alpha)}\left(\mathrm{ArcTan}\left[\frac{1-\alpha}{\sqrt{M-M^{*}}}\right] - \mathrm{ArcTan}\left[\frac{1-4p-\alpha}{\sqrt{M-M^{*}}}\right]\right) + \frac{1}{2}\left(\left(-2\,\mathrm{Log}[1-p] + \mathrm{Log}\left[1 + \frac{p}{M}(-1+2p+\alpha)\right]\right)\Big/(1+M+\alpha)\right) \qquad M^{*} = \frac{(1-\alpha)^2}{8} \tag{2}$$

Now, establishment is assured if, at time T_{crit}, $p > \hat{p} = \frac{(1-\alpha)}{2}$. Setting $p = \frac{(1-\alpha)}{2}$:

$$T_{\text{crit}} = \frac{2\,(3+\alpha)}{\sqrt{M-M^{*}}\,(1+M+\alpha)}\,\mathrm{ArcTan}\left[\frac{(1-\alpha)}{\sqrt{M-M^{*}}}\right] - \frac{\mathrm{Log}\left[\frac{1+\alpha}{2}\right]}{(1+M+\alpha)} \tag{3}$$

The left plot of Fig. B1 shows this critical time, as a function of the scaled migration rate, M. One might measure the total ‘cost’ of the introduction by MT. This declines to an asymptote for large M, indicating that the most efficient strategy is a short pulse that quickly raises the frequency above the critical threshold. However, MT is almost constant above $M \sim 0.1$, and so the precise timing of the introduction makes little difference (Fig. B1, middle). If the resident population is constant, N_0, then the number that need to be introduced to replace a fraction m is $\frac{m}{1-m} N_0$. With complete CI, $s_h = 1$, and so, this equals $\frac{M}{2-M}$: it becomes expensive to replace a large fraction of the population. When this effect is

included (Fig. B1, right), the most efficient strategy is at intermediate M. However, an instantaneous pulse is almost as efficient. The best guidance is that the introduction should be made as rapidly as is feasible, given practical constraints.

Figire B1: The left plot shows the minimum time needed for establishment, plotted against $M = \frac{2m}{s_h}$, as a function of α=0.1, 0.2, 0.3, 0.4, 0.5, 0.6 (bottom to top) (i.e., $\hat{p} = 0.45,\ 0.4,\ 0.35,\ 0.3,\ 0.25,\ 0.2$). The middle plot shows $M T_{\text{crit}} = m\, t_{\text{crit}}$, and the right plot $\frac{M}{2-M} T_{\text{crit}} = \frac{m}{2(1-m/s_h)} t_{\text{crit}}$

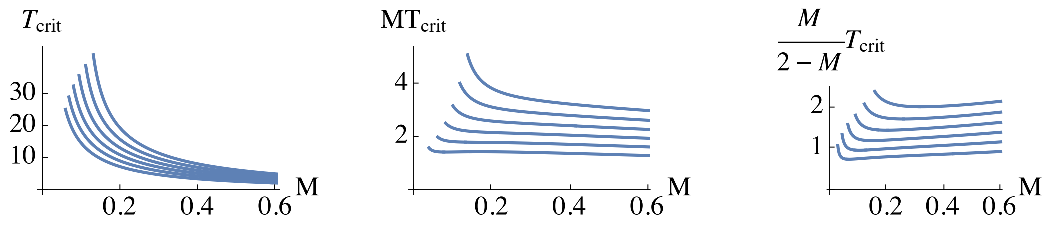

One dimension

We rescale Eqs. 2, 3 by setting time to $T = (s_h/2)\, t$ and distance to $X = x \sqrt{s_h} \big/ \sigma$; $M = \frac{2m}{s_h}$, $\alpha = 1 - 2\hat{p}$:

$$\frac{\partial p}{\partial T} = \frac{\partial^2 p}{\partial X^2} + p q\, (2p - 1 + \alpha) + M[X]\, q \tag{4}$$

With M=0, the wave will increase if the initial $p[X, 0]$ is "large enough". There is a critical bubble that lies on this theshold, which can be calculated explicitly in one dimension. Now, suppose that initially $p = 0$. How large must M be to ensure spread? This can be solved for special choices of the source, $M[X]$; below, we give the critical value for a point source, and for a "top hat" function, with a constant input within some region. We then give numerical results for the minimum time needed to ensure establishment, analogous to Fig. B1.

Point source

With a source $M\delta(X)q$, there is a boundary condition $p'[0, T] = \pm M q[0, T]/2$. Integrating Eq. 1 we have, at $X = 0$:

$$\frac{\partial p}{\partial X} = p \sqrt{q^2 - \alpha \left(1 - \frac{2}{3} p\right)} = \frac{M}{2} q \tag{5}$$

The function $2\, \frac{p}{q} \sqrt{q^2 - \alpha\left(1 - \frac{2}{3} p\right)}$ has an intermediate maximum, which gives the threshold value of Λ. Below this threshold, there are two solutions for p, one stable and one unstable:

$$M_{\text{crit}} = B \sqrt{1 - 4\alpha \frac{(1 - B/3)}{(2 - B)^2}}$$

$$\text{where } B = 2\left(1 - \frac{\alpha}{9}\right) - 2\alpha \left(1 + \frac{\alpha}{9}\right) \left(\frac{A}{2}\right)^{-1/3} - \frac{(4A)^{1/3}}{9}, \tag{6}$$

$$A = 243\,\alpha\left(1 + \frac{\alpha}{9} + \frac{2\,\alpha^2}{243} - \sqrt{1 + \frac{14}{81}\alpha + \frac{\alpha^2}{81}}\right)$$

In the original variables, the threshold scales as $\Lambda > g[\alpha]\,\frac{\sigma}{2}\sqrt{s_h}$ which has the dimensions $X T^{-1}$.

Top-hat source

Now, suppose that we have a source Mq within $-Y < X < Y$. Then, equilibrium is given by:

$$0 = \frac{\partial^2 p}{\partial X^2} + p\,(1-p)\,(2\,p - 1 + \alpha) + Mq \qquad (0 < X < Y)$$
$$0 = \frac{\partial^2 p}{\partial X^2} + p\,(1-p)\,(2\,p - 1 + \alpha) \qquad (Y < X) \tag{7}$$
$$\text{with } p'[0] = 0,\ \ p[Y_-] = p[Y_+],\ \ p'[Y_-] = p'[Y_+]$$

Integrating:

$$\left(\frac{\partial p}{\partial X}\right)^2 = h[p] \quad (Y < X)$$
$$\left(\frac{\partial p}{\partial X}\right)^2 = h[p] - M\,(p - p_Y)\,(2 - p_Y - p) \quad (0 < X < Y) \tag{8}$$
$$\text{where } h[p] = p^2\left((1-p)^2 - \alpha\left(1 - \frac{2}{3}p\right)\right)$$

The allele frequency at zero, p_0, is where $\partial p/\partial X = 0$, so that $0 = h[p_0] - M\,(p_0 - p_Y)\,(2 - p_0 - p_Y)$; this defines p_0 as a function of p_Y. We can obtain Y by integrating $\partial X/\partial p$. Hence, p_Y is given by:

$$Y = \int_{p_Y}^{p_0} \frac{dp}{\sqrt{h[p] - M\,(p - p_Y)\,(2 - p_0 - p_Y)}} \tag{9}$$

For given p_Y, there is a solution for Y, provided that $p_Y < \frac{2}{3}$; this has a maximum value of Y_{crit}, so that if $Y < Y_{crit}$, there will be two solutions. This maximum defines the threshold for spread. However, it is not straightforward to find this threshold analytically.

Consider the equation $0 = h[p_0] - M\,(p_0 - p_Y)\,(2 - p_0 - p_Y)$. For $M > (1-\alpha)^2/8$, there is only one root. For $M < (1-\alpha)^2/8$, there is one root if $p_Y > p_Y^*$.

$$p_0^* = \frac{1}{4}\left(1 - \alpha - \sqrt{A}\right)$$
$$p_Y^* = 1 - \frac{1}{4\sqrt{6\,M}}\sqrt{\left((1-\alpha)^3\,(3+\alpha) + 12\left(5 + 2\,\alpha + \alpha^2\right)M - 24\,M^2 - A\sqrt{A}\,(3+\alpha)\right)} \tag{10}$$

where $A = (1-\alpha)^2 - 8\,M$.

For $M > (1-\alpha)^2/8 = 0.02$, there is a single solution, with a peak which represents the maximum Y_{crit} consistent with a stable equilibrium. However, once M passes below the threshold, there is a singularity, and it appears that there can be a stable solution with extremely large Y. This threshold simply corresponds to M so low that it cannot cause a transition, even as $Y \to \infty$. Fig. B2 shows solutions to Eq. 10 over a range of α, $\hat{p}$; the maximum gives the critical spatial range, Y_{crit}, required for establishment. Table B1 gives this maximum, together with the total input, $2\,M Y_{crit}$. For given α, $\hat{p}$, there is an optimal migration rate and spatial extent that minimises $2\,M Y_{crit}$. These analytic results give the minimum input required for establishment. However, it would take an indefinitely long time for establish-

ment to be assured. If the input is more intense and/or over a wider spatial range, then it could persist for a shorter time. Results for a finite time, analogous to the island model (Fig. B1), require numerical solution. We give those in the next section, for two dimensions.

Figure B2. The value of Y against p_Y, for α=0.2, $\hat{p}$=0.2, and M= 0.02, 0.03, 0.04, 0.05, 0.06 (top to bottom). The maximum Y corresponds to the minimum size of the range, {−Y, Y} necessary for establishment, given a source M.

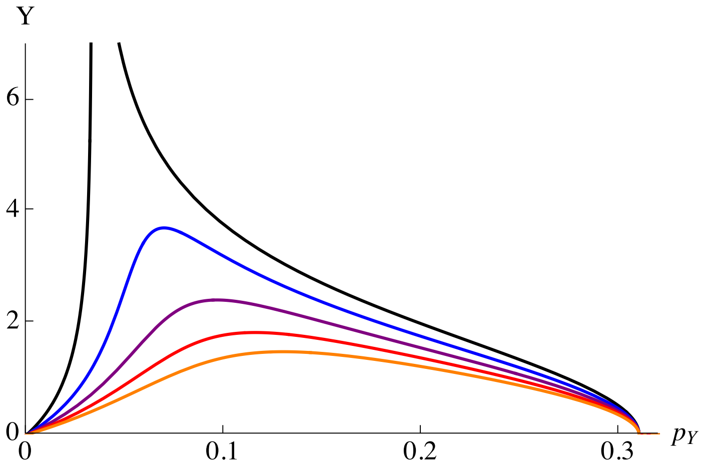

Table B1. Threshold Y_{crit} for varying α, M. p_Y, p_0 give the frequency at he edge of the source and at the centre, respectively. 2 MY gives the total rate of input. The last column, M_δ, gives the critical intensity of point source needed to allow establishment; it is the limit of the the total source 2 MY when M becomes large and its spatial extent, {−Y, Y} small.

α	$\hat{p}$	M	p_Y	p_0	Y_{crit}	2 MY_{crit}	M_δ
0.1	0.45	0.10			∞	∞	
		0.15	0.156	0.339074	2.86202	0.858606	
		0.2	0.230	0.397044	1.93251	0.773003	
		0.3	0.339	0.470151	1.2619	0.757142	
		0.4	0.408	0.512299	0.964945	0.771956	
		0.5	0.452	0.537759	0.789265	0.789265	
		0.6	0.483	0.554738	0.670762	0.804915	
		0.7	0.504	0.566513	0.584561	0.818385	0.956562
–––	–––	–––	–––	–––	–––	–––	
0.2	0.4	0.08			∞	∞	
		0.1	0.104	0.266342	4.13891	0.827782	
		0.15	0.189	0.333694	2.10228	0.630685	
		0.2	0.251	0.373722	1.50629	0.602516	
		0.3	0.328	0.418784	0.999828	0.599897	
		0.4	0.372	0.44259	0.760042	0.608034	0.684528
–––	–––	–––	–––	–––	–––	–––	
0.4	0.3	0.045			∞	∞	
		0.05	0.062	0.180264	6.71364	0.671364	
		0.1	0.163	0.25412	1.83648	0.367296	
		0.15	0.215	0.28246	1.17168	0.351503	
		0.2	0.245	0.297788	0.87301	0.349204	
		0.3	0.278	0.313796	0.584271	0.350562	
		0.4	0.295	0.322091	0.440908	0.352726	0.366544
–––	–––	–––	–––	–––	–––	–––	
0.6	0.2	0.02	0.033	0.100	∞		

0.05	0.116	0.168	1.83167	0.183167	
0.1	0.162	0.191	0.866468	0.173294	
0.15	0.179	0.199	0.575105	0.172531	
0.2	0.189	0.203	0.431589	0.172635	
0.3	0.198	0.208	0.288481	0.173089	
0.4	0.203	0.210	0.21682	0.173456	0.175184

Two dimensions

Using the same scalings as in one dimensions, we have:

$$\frac{\partial p}{\partial T} = \frac{\partial^2 p}{\partial R^2} + \frac{1}{R}\frac{\partial p}{\partial R} + p q\,(2p - 1 + \alpha) + M[R]\,q \tag{11}$$

We solve this equation numerically using NDSolve in *Mathematica*. For a given α, $\hat{p}$, there is a critical radius, R_{crit}, such that the wave will just establish if initially $p = 1$ within $R < R_{crit}$. We compare this initial pulse with a continuous source within the same radius, and sustained for time T; for given T, we find the minimum M required for establishment; this also gives the minimum T_{crit} needed for establishment with given M. As M increases above the threshold required for establishment from an indefinitely sustained source, T_{crit} decreases. Figure B3 summarises these results, in the same form as for Fig. B1, but just for $\alpha = 0.6$, $\hat{p} = 0.2$. As for the island model, the effort, measured by MT reaches an asymptote for large M, indicating that an immediate increase to high frequency is most efficient. If the increasing cost of raising frequency by introduction into a fixed native population is included, by using he measure $\frac{M}{2-M} T_{crit}$, then the most efficient scheme is to use $M \sim 0.3$ for $T_{crit} \sim 1$ - which is still an extremely short time if $s_h = 1$.

Figure B3. On the left, the time for which a source needs to be sustained, plotted against source strength, M, given optimal initial radius. The vertical line is the source strength needed for establishment, if sustained indefinitely. The middle plot shows the total amount, $M T_{crit} = \frac{m}{2} t_{crit}$ that is needed, as a function of source strength. The right plot shows $\frac{M}{2-M} T_{crit} = \frac{m}{2(1-m/s_h)} t_{crit}$, which is the total number that need to be introduced, relative to a constant native population, if $s_h = 1$. $R_{crit} = 5.19$, $\alpha = 0.6$, $\hat{p} = 0.2$.

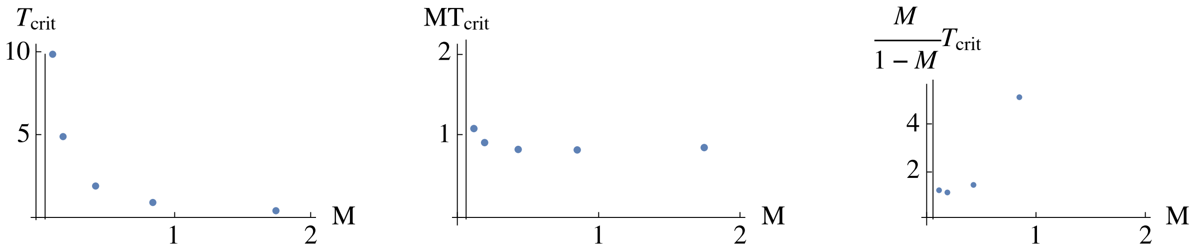

Supplement: 3 [file NIHMS868121-supplement-3.pdf]
